# Lewis Acidic Ionic Liquids

**DOI:** 10.1007/s41061-017-0166-z

**Published:** 2017-08-21

**Authors:** Lucy C. Brown, James M. Hogg, Małgorzata Swadźba-Kwaśny

**Affiliations:** School of Chemistry and Chemical Engineering, The Queen’s University of Belfast, David Keir Building, Stranmillis Road, Belfast, BT9 5AG UK

**Keywords:** Lewis acidity, Halometallate ionic liquids, Liquid coordination complexes, Solvate ionic liquids, Borenium cations

## Abstract

Until very recently, the term Lewis acidic ionic liquids (ILs) was nearly synonymous with halometallate ILs, with a strong focus on chloroaluminate(III) systems. The first part of this review covers the historical context in which these were developed, speciation of a range of halometallate ionic liquids, attempts to quantify their Lewis acidity, and selected recent applications: in industrial alkylation processes, in supported systems (SILPs/SCILLs) and in inorganic synthesis. In the last decade, interesting alternatives to halometallate ILs have emerged, which can be divided into two sub-sections: (1) liquid coordination complexes (LCCs), still based on halometallate species, but less expensive and more diverse than halometallate ionic liquids, and (2) ILs with main-group Lewis acidic cations. The two following sections cover these new liquid Lewis acids, also highlighting speciation studies, Lewis acidity measurements, and applications.

## Introduction

The development and applications of Lewis acids hold an important place in chemical research. In industrial processes, heterogeneous Lewis acids are dominant, from simple metal halides to metal oxides with Lewis acidic sites (alumina, zirconia, titania) [1, 2]. Lewis acids, such as BF_3_ or AlCl_3_, are also combined with Brønsted acids to yield Brønsted superacidic catalysts. In organic synthesis, a wide variety of Lewis acids are used, both in stoichiometric and catalytic quantities, with acidic metal centers varying from alkali metals (Li, Na), through to group 13 elements (Al^III^, Ga^III^, In^III^), to Zn^II^, Sn^II^ and Sn^IV^, Hf^IV^ and lanthanides [3]. In addition to simple halides, metal triflates and bistriflamides are also used, as well as organometallic Lewis acids, with an increasing focus on water-stable Lewis acids [4]. Elaborate chiral ligands are used for asymmetric reactions [5]. In main-group chemistry, frustrated Lewis pairs, which are combinations of Lewis acids and bases prevented from forming an adduct by steric hindrance [6], have recently opened up the field of metal-free catalysis and small molecule activation [7]. In this context, the archetypical Lewis acid is B(C_6_F_5_)_3_, though a plethora of charge-neutral and cationic Lewis acids, based predominantly on boron, but also on phosphorus, silicon and main-group metals, have been synthesized [8–10].

Against this backdrop, the chemistry of Lewis acidic ionic liquids appears structurally monotonous, with a strong focus on halometallate Lewis acidic anions—in particular chloroaluminate(III) ILs. These were the first group of ionic liquids to capture the attention of chemists across disciplines, and remained the center of research efforts until ca. 2000 [11, 12]. Afterwards, the spotlight has shifted to ‘air- and water-stable’ systems [13], but research on Lewis acidic halometallate ILs has been under continuous development, which was reviewed in 2014 by Estager et al. [14]. Recent years have been marked by: (1) the maturing of chloroaluminate(III) ILs in catalysis, expressed in engineering advances and the announcement of several full-scale industrial processes, (2) the development of new ionic liquid-like systems, based on metal chloride eutectics (liquid coordination complexes), and (3) the naissance of ILs with Lewis acidic cations, with hopefully a lot of inspiring chemistry still to come. All three families of Lewis acidic ionic liquids will be discussed, including the historical context, speciation studies, quantification of acidity, and examples of applications. Abbreviations used throughout the text are listed in Table 1.

**Table 1:** Abbreviations used in this work (where *n* = alkyl chain length)

Notation	Full name
[C_*n*_mim]^+^	1-Alkyl-3-methylimidazolium cation
[P_*nnnn*_]^+^	Tetraalkylphosphonium cation
[N_*nnnn*_]^+^	Tetraalkylammonium cation
MX_*m*_	Metal halide
[OTf]^−^	Triflate anion
[NTf_2_]^−^	*Bis*(trifluoromethanesulfonyl)imide anion, [N(SO_2_CF_3_)_2_]^−^
[BETI]^−^	*Bis*(trifluoroethanesulfonyl)imide anion, [N(SO_2_C_2_F_5_)_2_]^−^
G*n*	Glycol ether (glyme), with *n* + 1 oxygens in its structure
RON	Research octane number
SILP	Supported ionic liquid phase
SCILL	Solid catalyst with ionic liquid layer
TEPO	Triethylphosphine oxide

## Chlorometallate Ionic Liquids

### Historical Context

**Fig. 1:**
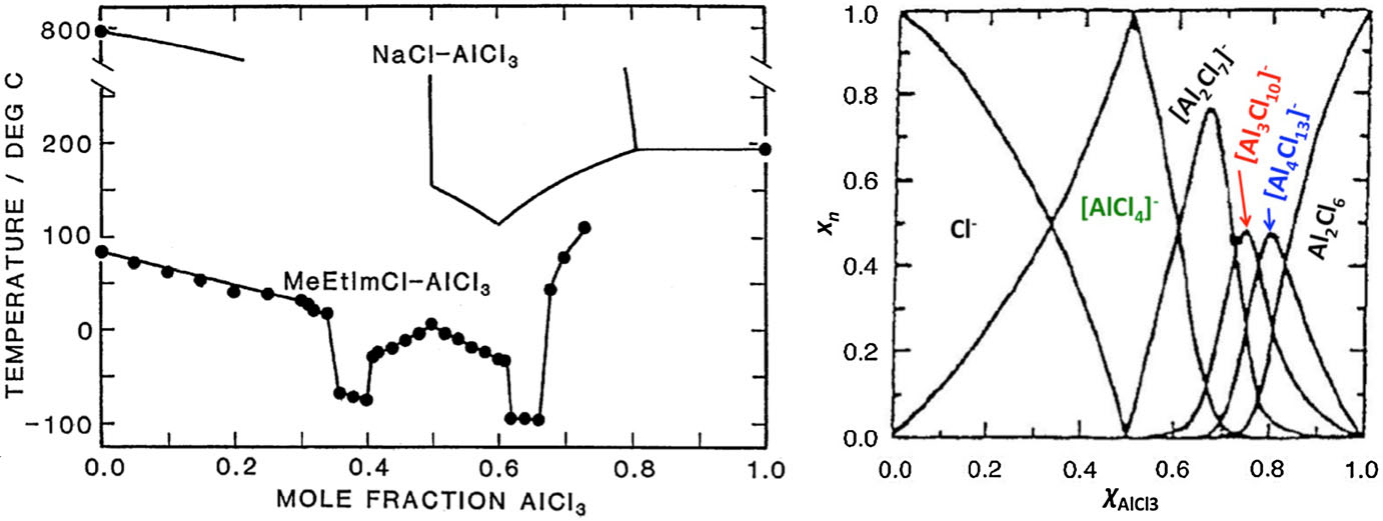
*Left* Comparison of phase diagrams of NaCl–AlCl_3_ and 1-ethyl-3-methylimidazolium chloride-AlCl_3_ [15]. *Right* Concentration of anionic species in the [C_2_mim]Cl–AlCl_3_ system, at 200 °C, calculated from a thermodynamic model. (From [14], adapted from [18])

The study of modern ionic liquids originated from high-temperature inorganic molten salts (studied as heat transfer fluids and electrolytes), which lead to lower melting organic salts, especially chloroaluminate(III) ILs (Fig. 1, left), based on [AlCl_4_]^−^ and [Al_2_Cl_7_]^−^ anions [15]. In parallel, mechanistic studies on AlCl_3_-promoted Friedel–Crafts chemistry revealed [AlCl_4_]^−^ and [Al_2_Cl_7_]^−^ anions to balance the charge of the intermediate, a protonated toluenium cation [12, 16]. This naturally led to studies of Friedel–Crafts chemistry in the [C_2_mim]Cl–AlCl_3_ system [17], which may be considered the starting point of Lewis acid catalysis in chlorometallate ILs, with over 400 citations to date.

Boon’s work was preceded—by over a decade—by Parshall, who in 1972 used organic molten salts, [N_2222_][SnCl_3_] and [N_2222_][GeCl_3_], as solvents for PtCl_2_ [19]. They also acted as Lewis acidic co-catalysts in hydrogenation and other Pt-catalyzed reactions, through the formation of Pt active species, such as [Pt(SnCl_3_)_5_]^3−^. It was a visionary paper, but initially went almost unnoticed, and was only sparsely cited until its re-discovery in the community around 2000. This possibly asserted the dominance of chloroaluminate(III) ILs as the archetypical Lewis acidic ionic liquids.

### Speciation

Halometallate ILs are synthesized by the reaction of a metal halide with an organic halide salt, at various molar ratios (Eq. 1) [14]. The composition of the ionic liquid is typically reported as the molar ratio of metal halide, *χ*_MX*m*_ (see Eq. 2 and Fig. 1).

1 $$\left[ {\text{cation}} \right]X + nMX_{m} \to \left[ {\text{cation}} \right]\left[ {M_{n} X_{n \times m + 1} } \right]$$

2 $$\chi_{{{\text{MX}}m}} = {{n\left( {{\text{MX}}_{m} } \right)} \mathord{\left/ {\vphantom {{n\left( {{\text{MX}}_{m} } \right)} {\varSigma \left( n \right)}}} \right. \kern-0pt} {\varSigma \left( n \right)}}$$

The range of *χ*_MX*m*_ values that yield homogenous ILs varies, depending on the metal and the halide. Within this range, different halometallate anions may be formed, depending on the composition. More often than not, several anions in a dynamic equilibrium with each other are present.

#### Chloroaluminate(III) Ionic Liquids

Complex anionic speciation of halometallate ionic liquids is reflected in the exemplary phase diagram for chloroaluminate(III) ILs (Fig. 1). It is the speciation that dictates their physical properties and chemical behavior, including Lewis acidity. Organic cations are considered to affect physical properties of halometallate ILs, such as viscosity, density, or melting points, but typically have little influence on the anionic speciation, and thus on Lewis acidity [14]. Anionic speciation in chloroaluminate(III) systems was confirmed using multiple direct techniques, such as ^27^Al NMR spectroscopy [20], Raman spectroscopy [21], and supporting techniques, such as phase diagrams [22]. In-depth discussion of these techniques is beyond the scope of this work, but extensive primary literature exists, as well as older reviews [11, 23] and a recent review by our group [14].

Concentration of anionic chlorometallate(III) species as a function of composition, calculated from a thermodynamic model for [C_2_mim]Cl–AlCl_3_ (Fig. 1, right), is concurrent with phase diagram for the same system (Fig. 1, left). For the *χ*_AlCl3_ = 0.50 composition (equimolar), there is only one anion present, [AlCl_4_]^−^; the peritectic point in the phase diagram (Fig. 1, left) indicates the formation of a compound, [C_2_mim][AlCl_4_]. For all other compositions, multiple equilibrated species are present. With excess of [C_2_mim]Cl (*χ*_AlCl3_ < 0.50), free chloride is in equilibrium with [AlCl_4_]^−^. For an excess of AlCl_3_, up to *χ*_AlCl3_ = 0.67 (2:1 ratio), dinuclear [Al_2_Cl_7_]^−^ and monomeric [AlCl_4_]^−^ are equilibrated. In both cases, this is reflected in the formation of very low-melting, eutectic compositions. Finally, for large excess of AlCl_3_ (*χ*_AlCl3_ > 0.67), oligomeric anions were postulated (Fig. 1, right), but these exist in equilibrium with solid aluminium(III) chloride. Therefore, homogenous ionic liquids are only formed for compositions *χ*_AlCl3_ ≤ 0.67, and the slightest excess of AlCl_3_ added above this ratio results in cloudiness of otherwise clear liquid. The main anionic equilibrium in chloroaluminate(III) ILs is shown in Eq. 3.

3 $$2\left[ {{\text{AlCl}}_{4} } \right]^{ - } \rightleftarrows \left[ {{\text{Al}}_{2} {\text{Cl}}_{7} } \right]^{ - } + {\text{ Cl}}^{ - }$$

**Scheme 1:**
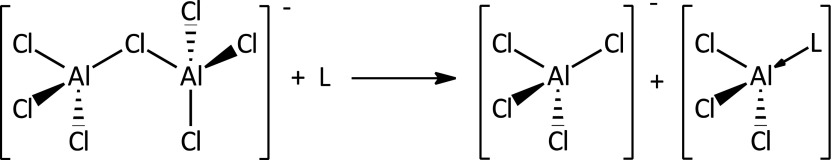
Reaction of the Lewis acidic anion [Al_2_Cl_7_]^−^, with a base L

There is a crucial link between speciation and Lewis acidity. The four chloride ligands around the aluminium center in [AlCl_4_]^−^ effectively prevent other ligands from coordinating to form five- and six-coordinate complexes, and thus render the anion neutral. In the vast majority of Lewis acid-catalyzed reactions, ILs with this anion have no activity. Halide anions are Lewis basic [24, 25], therefore the *χ*_AlCl3_ < 0.50 compositions are Lewis basic. The dimeric [Al_2_Cl_7_]^−^ anion is Lewis acidic [26, 27], however, it is worth noting that also here the chloride species pose enough steric hindrance to prevent the formation of higher coordination. However, it is a ‘latent’ Lewis acid [28] due to an easily broken chloride bridge, which reacts with a base following Scheme 1.

There are many applications of chloroaluminate(III) ILs, such as electrode positions, which are not directly related to their Lewis acidity. The primary use of chloroaluminates(III) as Lewis acids is in catalysis; they are used directly as Lewis acidic catalysis (for example Diels–Alder reactions), or they react with residual water/protic additives to generate a Brønsted superacid (carbocationic reactions, e.g., isomerization) [11, 14]. Chloroaluminates(III) have also been used as solvents and Lewis acidic co-catalysts in reactions catalyzed by transition metal complexes [29]. A more recent addition to Lewis acidic applications of chloroaluminate(III) ILs is the area of inorganic synthesis, where they were used in multiple roles of solvent, reactant, and base scavenger [30].

Although they are the most common family of halometallate ionic liquids, the speciation of chloroaluminate(III) ILs can never be considered as the ‘benchmark’; numerous studies have explicitly shown that anionic speciation is unique for each metal, and must be investigated for every system anew, before we can understand its chemistry [14].

For known chlorometallate ILs, dominant species as a function of composition are listed in Table 2 [14]. The common denominator is that at low (basic) metal chloride loadings, the anionic species are a mixture of free chloride and saturated chlorometallate anions. As metal chloride loading increases, at a certain *χ*_MXm_ value there is no free chloride anions, as they are bound to the metal, forming coordinatively saturated (neutral) halometallate complex—typically this is associated with the compound formation point in a phase diagram. Upon further increasing of metal chloride content, there are three scenarios possible: (1) oligomeric (‘latent’ Lewis acidic) chlorometallate anions are formed, (2) monomeric, coordinatively unsaturated (Lewis acidic) chlorometallate species exist, and (3) excess metal chloride precipitates.

**Table 2:** Anionic speciation of known chlorometallate ILs, in the liquid state, as a function of composition. Adapted and updated from [14]

*χ*_MXx_							Analysis methods
	0.25	0.33	0.50	0.60	0.67	0.75	
Ti(IV)		[TiCl_6_]^2−^			[Ti_2_Cl_9_]^−^		IR, Raman [31]
Zr(IV)	Cl^−^; [ZrCl_6_]^2−^*	[ZrCl_6_]^2−^*	[Zr_2_Cl_10_]^2−^*		[Zr_2_Cl_9_]^−^*		DSC [32], crystallography
Hf(IV)	Cl^−^; [HfCl_6_]^2−^*	[HfCl_6_]^2−^*	[Hf_2_Cl_10_]^2−^*		[Hf_2_Cl_9_]^−^*		DSC [32]
Nb(V)	Cl^−^; [NbCl_6_]^−^		[NbCl_6_]^−^	[NbCl_6_]^−^; Nb_2_Cl_10_			Raman [33]
Ln(III)**	[LnCl_6_]^3−^						EXAFS, crystallography [34]
Mn(II)		[MnCl_4_]^2−^	[MnCl_3_] ^−^*				Raman, IR [35], EXAFS [36]
Fe(II)		[FeCl_4_]^2−^		[FeCl_4_]^2−^; FeCl_2_↓			Raman, XPS [37, 38]
Fe(III)	Cl^−^; [FeCl_4_]^−^		[FeCl_4_]^−^	[FeCl_4_]^−^; [Fe_2_Cl_7_]^−^	[FeCl_4_]^−^; [Fe_2_Cl_7_]^−^; FeCl_3_↓		Raman, IR, XPS [37–39]
Co(II)	Cl^−^; [CoCl_4_]^2−^	[CoCl_4_]^2−^	[CoCl_2_(CoCl_4_)_2_]^4−^; [Co(CoCl_4_)_3_]^4−^*	[CoCl_2_(CoCl_4_)_2_]^4−^; [Co(CoCl_4_)_3_]^4−^; CoCl_2_↓*			UV–Vis [40]
Ni(II)	Cl^−^; [NiCl_4_]^2−^	[NiCl_4_]^2−^	[NiCl_4_]^2−^; NiCl_2_↓				Crystallography [36], EXAFS [41, 42]
Cu(I)	Cl^−^; [CuCl_(2+n)_]^(1−*n*)−^		[CuCl_2_]^−^		[Cu_2_Cl_3_]^−^		IR, Raman [43], viscometry [44]
Cu(II)		[CuCl_4_]^2−^	[Cu_2_Cl_6_]^2−^*				UV–VIS, EXAFS [45, 46]
Au(III)	Cl^−^; [AuCl_4_]^−^		[AuCl_4_]^−^	[AuCl_4_]^−^; AuCl_3_↓			Raman, ab initio [47]
Zn(II)	Cl^−^; [ZnCl_4_]^2−^	[ZnCl_4_]^2−^	[Zn_2_Cl_6_]^2−^	[Zn_2_Cl_6_]^2−^; [Zn_3_Cl_8_]^2−^	[Zn_3_Cl_8_]^2−^	[Zn_4_Cl_10_]^2−^	Raman, crystal structure, EXAFS [48]
Cd(II)		[CdCl_4_]^2−^*	[Cd_2_Cl_6_]^2−^*				Crystal structure [49]
Hg(II)	Cl^−^; [HgCl_4_]^2−^	[HgCl_4_]^2−^	[Hg_2_Cl_6_]^2−^	[Hg_3_Cl_8_]^−^	[Hg_2_Cl_5_]^−^		Crystal structure [50], ^199^Hg NMR, Raman [51], EXAFS [52]
Al(III)	Cl^−^; [AlCl_4_]^−^		[AlCl_4_]^−^	[AlCl_4_]^−^; [Al_2_Cl_7_]^−^	[Al_2_Cl_7_]^−^	[Al_x_Cl_y_]^−^; AlCl_3_↓	Raman [21], ^27^Al NMR [20]
Ga(III)	Cl^−^; [GaCl_4_]^−^		[GaCl_4_]^−^	[GaCl_4_]^−^; [Ga_2_Cl_7_]^−^	[Ga_2_Cl_7_]^−^	[Ga_3_Cl_10_]^−^	Raman [53], ^71^Ga NMR, EXAFS [53–55]
In(III)	[InCl_6_]^3−^	[InCl_5_]^2−^	[InCl_4_]^−^	[InCl_4_]^−^; InCl_3_↓			Raman, ^115^In NMR, XPS [55–57]
Sn(II)	Cl^−^; [SnCl_3_]^−^		[SnCl_3_]^−^	[SnCl_3_]^−^; [Sn_2_Cl_5_]^−^	[SnCl_3_]^−^; [Sn_2_Cl_5_]^−^; SnCl_2_↓		Raman, XPS [58]
Pb(II)	Cl^−^; [PbCl_4_]^2−^	[PbCl_4_]^2−^	[PbCl_4_]^2−^; [PbCl_3_]-; PbCl_2_↓				Raman, ^207^Pb NMR [59]

Dominant anions in given compositions are listed, along with major techniques used to confirm the speciation

* Speciation assumed based on stoichiometry, solid-state studies or indirect techniques, such as DSC

** Ln = La, Ce, Pr, Nd, Sm, Eu, Gd, Tb, Dy, Ho, Er, Tm, Yb, Lu

It is crucial to note that, for asserting correct assignment of species present in each composition, a range of techniques should be used, preferably applied directly to neat ionic liquid: multinuclear NMR spectroscopy, IR, and Raman spectroscopies, XPS. In addition to these, phase diagrams are indispensable.

#### Chlorogallate(III) Ionic Liquids

Gallium follows aluminium in Group 13 of the periodic table, and indeed follows a very similar speciation pattern. However, whereas AlCl_3_ precipitates from *χ*_AlCl3_ > 0.67 compositions, chlorogallate(III) ILs remain homogenous at least up to *χ*_GaCl3_ = 0.75, and even beyond this composition, forming very reactive and strongly Lewis acidic anions, such as [Ga_3_Cl_10_]^−^ [53–55]. Despite high catalytic activity, far fewer catalytic applications are described when compared to chloroaluminates(III); most likely due to the higher price of GaCl_3_ vs. AlCl_3_. Nevertheless, they have been used in acetalization of aldehydes [60], oligomerization of 1-decene [61], alkylation of isobutane with butane [53], and Bayer–Villiger oxidation with H_2_O_2_ [62]. In general, enhanced moisture stability compared to chloroaluminates(III) was noted, which led to some advantages, such as: good recyclability in refinery alkylation [53], decreased isomerization in oligomerization of 1-decene [61], and the relative stability of the active intermediate, [GaCl_3_OH]^−^, in Bayer–Villiger oxidation [62].

#### Chloroindate(III) Ionic Liquids

Also belonging to Group 13, chloroindate(III) anions are not capable of formation of oligomeric species, but InCl_3_ precipitates readily at *χ*_InCl3_ > 0.50. However, in contrast to smaller icosagens, indium(III) can accommodate up to six chlorides, forming a compound at *χ*_InCl3_ = 0.25, with [InCl_6_]^3−^ (Eq. 4) [55–57]. At *χ*_InCl3_ = 0.33, some quantities of [InCl_5_]^2−^ may be forming, although this is not explicitly proven, and the equilibrium in Eq. 4, may be very strongly shifted to the right.

4 $$\left[ {\text{cation}} \right]{\text{Cl }} + 3{\text{InCl}}_{3} \to \left[ {\text{cation}} \right]_{3} \left[ {{\text{InCl}}_{6} } \right]$$

5 $$4{\text{Cl}}^{ - } + 2{\text{InCl}}_{3} \to 2\left[ {{\text{InCl}}_{5} } \right]^{2 - } \rightleftarrows \left[ {{\text{InCl}}_{6} } \right]^{3 - } + \, \left[ {{\text{InCl}}_{ 4} } \right]^{ - }$$

Finally, like other icosagenes, chloroindate(III) ILs at *χ*_InCl3_ = 0.50 form [InCl_4_]^−^. However, this composition is not neutral—[InCl_4_]^−^ is coordinatively unsaturated, and reacts with a base as a mild Lewis acid (Eq. 6).

6 $$\left[ {{\text{InCl}}_{4} } \right]^{ - } + \, 2{\text{L}} \to \left[ {{\text{InCl}}_{4} {\text{L}}_{2} } \right]^{ - }$$

Chloroindate(III) ionic liquids are hydrolytically stable [63] and mildly Lewis acidic [27], which contrasts with systems based on lighter icosagenes, and opens up new application routes, such as: transesterification (biodiesel synthesis) [64], alkylation of phenols or catechols with alcohols [63], as well as for the protection of alcohols, followed by their conversion to acetates and trimethylsilyl ethers [65]. Noteworthy, in some instances compositions with excess of InCl_3_ are used (*χ*_InCl3_ > 0.50), even though they form a suspension/paste, rather than the ionic liquid [66].

#### Chloroferrate(III) Ionic Liquids

Ionic liquids based on iron(III) chloride form neutral [FeCl_4_]^−^ at *χ*_FeCl3_ = 0.50, and both [FeCl_4_]^−^ and the Lewis acidic [Fe_2_Cl_7_]^−^ can be detected at *χ*_FeCl3_ = 0.60 [37, 38]. However, analogy with chloroaluminate(III) systems proves to be deceitful, as FeCl_3_ has been reported to precipitate at *χ*_FeCl3_ > 0.60 compositions [67]. Consequently, attaining homogenous ILs at *χ*_FeCl3_ = 0.67 is impossible.

Catalytic applications of chloroferrates(III) were reviewed in 2011 by Bica and co-workers [68]. Their mild Lewis acidity is combined with interesting physical properties: thermomorphic behavior with water (thermally induced demixing) [39, 69] and paramagnetic behavior [70, 71], which were both suggested as routes for facile separation and recycling. Notably, despite their rather hydrophobic nature, chloroferrates(III) hydrolyze slowly in the presence of moisture [69].

#### Chlorozincate(III) Ionic Liquids

These ionic liquids are typically more viscous and higher melting than other halometallates, as chlorozincate(II) form doubly negatively charged anions [25, 33, 48, 72, 73]. A neutral compound is formed at *χ*_ZnCl2_ = 0.33, with the [ZnCl_4_]^2−^ anion (Eq. 7), and all *χ*_ZnCl2_ > 0.33 compositions are Lewis acidic. Several oligomeric chlorozincate(II) anions exist: [Zn_2_Cl_6_]^2−^, [Zn_3_Cl_8_]^2−^ and [Zn_4_Cl_10_]^2−^ have all been postulated. Reaction of a base with an oligomeric chlorozincate anion, which is a ‘latent’ Lewis acid, can be envisaged as shown in Eq. 8.

7 $$\left[ {\text{cation}} \right]{\text{Cl }} + 2{\text{ZnCl}}_{2} \to \left[ {\text{cation}} \right]_{2} \left[ {{\text{ZnCl}}_{4} } \right]$$

8 $$\left[ {{\text{Zn}}_{2} {\text{Cl}}_{6} } \right]^{2 - } + \, 2{\text{L}} \to \left[ {{\text{ZnCl}}_{4} } \right]^{2 - } + \left[ {{\text{ZnCl}}_{2} {\text{L}}_{2} } \right]$$

It is important to note that, based on early mass spectrometry studies, some authors postulated that chlorozincate(II) ILs adopt speciation analogous to that of chloroaluminate(III) ILs, i.e., to form [ZnCl_3_]^−^ and [Zn_2_Cl_5_]^−^ anions. This assumption is still very prevalent in the literature, however, there are a number of arguments against it:

- tricoordinate zinc complexes are very few and only found in extremely sterically hindered environments; otherwise, tetracoordinate geometry is strongly preferred;
- not a single crystal structure exists with [ZnCl_3_]^−^ or [Zn_2_Cl_5_]^−^ anions, in contrast to plethora of structures with tetracoordinate chlorozincate(II) anions;
- physical properties of chlorozincate ILs (high viscosity) are consistent with doubly charged anions;
- all spectroscopic techniques applied directly to the liquid phase (XPS, EXAFS, Raman spectroscopy) support the existence of zinc(II) in tetracoordinate environment;
- MS is a gas-phase technique, which may give erroneous results for the speciation of liquid with dense network of Coulombic and hydrogen bonding interactions.

In terms of catalysis, chlorozincate(II) ILs are appreciated for their low cost and stability against moisture, which leads to robust and recyclable catalysts [74, 75]. They find uses where stronger acids would either hydrolyze, or destroy the reactants, such as *O*-acylation of cellulose [76]. In an interesting recent application, chlorozincate(II) ionic liquids diluted with [C_4_mim][BF_4_] were used as a liquid support and co-catalyst in chemoselective reduction of heteroarenes, promoted by Rh nanoparticles [77].

#### Chlorostannate(II) Ionic Liquids

**Fig. 2:**
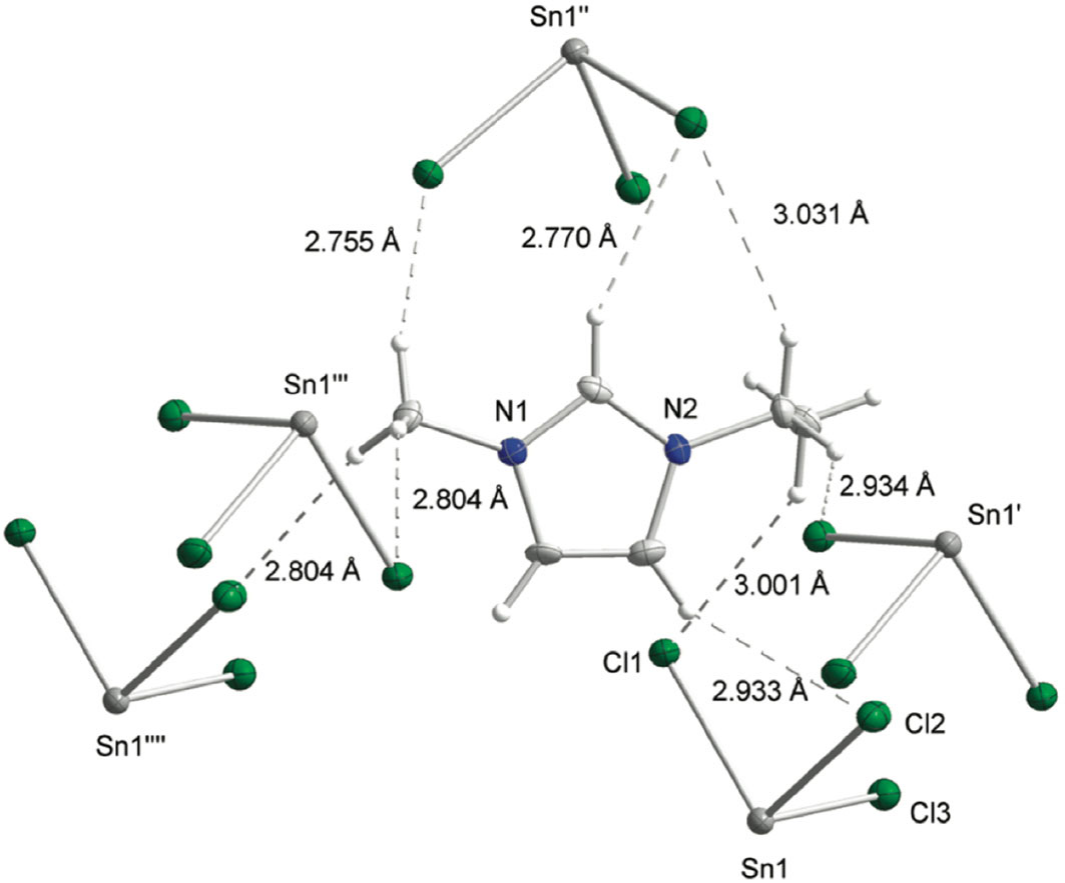
Crystal structure of [C_2_mim][SnCl_3_], showing the hydrogen-bonding interactions of the 1-ethyl-3-methylimidazolium cation and the [SnCl_3_]^−^ anion [58]

Anionic speciation of chlorostannate(II) ILs is worth contrasting with chlorozincates(II); chlorostannates(II) do form tricoordinate [SnCl_3_]^−^ and [Sn_2_Cl_5_]^−^ anions (Eqs. 9, 10), with a free electron pair occupying the fourth coordination position around the tin(II) atom in pseudo-tetrahedral trigonal pyramidal geometry (Fig. 2) [58]. In addition, although known in crystalline state, chlorostannate(II) ILs do not form [SnCl_4_]^2−^ in the liquid state.

9 $$\left[ {\text{cation}} \right]{\text{Cl }} + {\text{SnCl}}_{2} \to \left[ {\text{cation}} \right]\left[ {{\text{SnCl}}_{3} } \right]$$

10 $$\left[ {\text{cation}} \right]{\text{Cl }} + \, 2{\text{SnCl}}_{2} \to \left[ {\text{cation}} \right]\left[ {{\text{Sn}}_{2} {\text{Cl}}_{5} } \right]$$

Chlorostannate(II) anions are amphoteric, which is expressed in moderately high donor and acceptor properties of chlorostannate(II) ionic liquids [24]. In 1972, Parshall used chlorostannate(II) ILs (then called fused salts) as solvents for PdCl_2_, and Lewis basic co-catalysts for hydrogenation, isomerization, hydroformylation and carboalkoxyaltion of olefins [19]. The mechanism of a similar process, Pt^II^-promoted hydroformylation in chlorostannate ILs, was studied over thirty years later by van Eldik and co-workers, who explicitly shown the [SnCl_3_]^−^ coordinating to the platinum center in the active form of the catalyst [78]. On the other side of the spectrum, Abbott and co-workers used chlorostannate(II) ILs as Lewis acidic catalysts for Diels–Alder reaction, in which they were less active than chlorozincate(II) analogues [75]. Zhang and co-workers used them for the preparation of highly isotactic and optically pure l-lactide, remarking on their low price, good moisture stability and tunability of the acidity by altering the *χ*_SnCl2_ value [79]. However, it must be noted that chlorostannate(II) anions are not entirely stable towards atmospheric oxygen, and upon prolonged exposure oxidize to [SnCl_6_]^2−^, especially in the presence of free chlorides [19, 58].

### Acidity Measurements

The strength of interaction of a Lewis acid–base pair depends on the size, shape and relative energies of the LUMO of the acid and the HOMO of the base, in addition to steric effects from both components [80]. Consequently, the strength of a Lewis acid depends on the base it is interacting with. This is in contrast to Brønsted acids, which are always quantified with respect to the same species: the proton. As such, there is no absolute, direct method of quantifying the strength of a Lewis acid. Rather, there is a number of established Lewis acidity scales, whereby a basic probe molecule or ion is used, and the strength interaction between the probe and the acid is quantified by: (1) computational calculations [81, 82], (2) a physical measurement [83–85], or (3) by reaction rate of a model reaction catalyzed by the studied acid [86, 87]. Discussed below are selected methods used for quantifying Lewis acidity in ionic liquids.

#### Gutmann Acceptor Number: NMR Spectroscopic Method

The Gutmann AN approach, initially developed to study donor/acceptor properties of solvents and then expanded to liquid acids [84, 88], has been the first method used to quantify Lewis acidity of ILs [26, 89]. In the Gutmann Acceptor Number (AN) method, triethylphosphine oxide (TEPO) is used as the ^31^P NMR spectroscopic probe. Advantages of TEPO include good sensitivity of the ^31^P nucleus (naturally 100% abundant, spin 1/2, highly sensitive to its environment), relative stability towards strong acids, low steric hindrance and good solubility in a wide variety of solvents [90]. Coordination of the phosphine oxide to a Lewis acid induces change in the ^31^P NMR chemical shift. The scale was arbitrarily defined based upon the ^31^P NMR chemical shift of the probe molecule in hexane (AN = 0) and SbCl_5_ in 1,2-dichloroethane (AN = 100) [91]. To eliminate the influence of concentration, ^31^P NMR spectra of TEPO at several small concentrations are recorded, and the chemical shift is extrapolated to infinite dilution (*δ*_inf_), which is then normalized with respect to infinite dilution of TEPO in hexane (Δ*δ*_inf_). AN values are calculated from Eq. 11.

11 $${\text{AN}} = 2.348 \times \Delta \delta_{\inf }$$

Beckett et al. proposed a modified method, in which a constant mass of TEPO is dissolved in a constant volume of sample, without extrapolating to infinite dilution [85]. The Gutmann–Beckett method gained popularity for the study of molecular Lewis acids; however, it was observed that for complex equilibria found in Lewis acidic ILs, even small changes in concentration affect the chemical shift, therefore extrapolating to infinite dilution is recommended for the highest reproducibility [27]. Although TEPO decomposition was never reported for ILs, some extremely strong Lewis acids have been known to decompose it [92].

The scale is very versatile, it was used to study not only strong Lewis acids, but to distinguish between subtle differences in acceptor properties of ILs cations [24, 93].

Gutmann AN was firstly used by Osteryoung and co-workers, to quantify the acidity of chloroaluminate(III) ILs [26]. AN = 98.2 was recorded for a Lewis basic system (*χ*_AlCl3_ = 0.45) and AN = 103.2 was reported for the most Lewis acidic one (*χ*_AlCl3_ = 0.67). For different cations the discrepancy between two systems at the same composition (*χ*_AlCl3_ = 0.55) was marginal, AN = 98.2 and 98.6 recorded for [C_4_py]^+^ and [C_2_mim]^+^, respectively. Compared to hexane (AN = 0) and water (AN = 54.8), all compositions appeared to be strong acids, which is clearly false. It was demonstrated that, due to high oxophilicity of aluminium, ligand displacement took place (Eq. 12).

12 $$\left[ {{\text{AlCl}}_{4} } \right]^{ - } + {\text{TEPO}} \to \left[ {{\text{AlCl}}_{3} \left( {\text{TEPO}} \right)} \right] + {\text{Cl}}^{ - }$$

In their subsequent work, Osteryoung and co-workers were able to fit equations to ^31^P NMR chemical shifts recorded for TEPO in chloroaluminate(III) ILs (for *χ*_AlCl3_ ≤ 0.50), and relate them to the concentration of [AlCl_4_]^−^ and [Al_2_Cl_7_]^−^ [94]. This was possible for neat chloroaluminate ionic liquids, and for those where acidity was buffered with alkali metal salts (Eq. 13).

13 $$\left[ {{\text{Al}}_{2} {\text{Cl}}_{7} } \right]^{ - } + {\text{ MCl}}_{(s)} \to 2\left[ {{\text{AlCl}}_{4} } \right]^{ - } + {\text{M}}^{ + } \left( {{\text{M}} = {\text{Li}},{\text{ Na}},{\text{ K}}} \right)$$

**Fig. 3:**
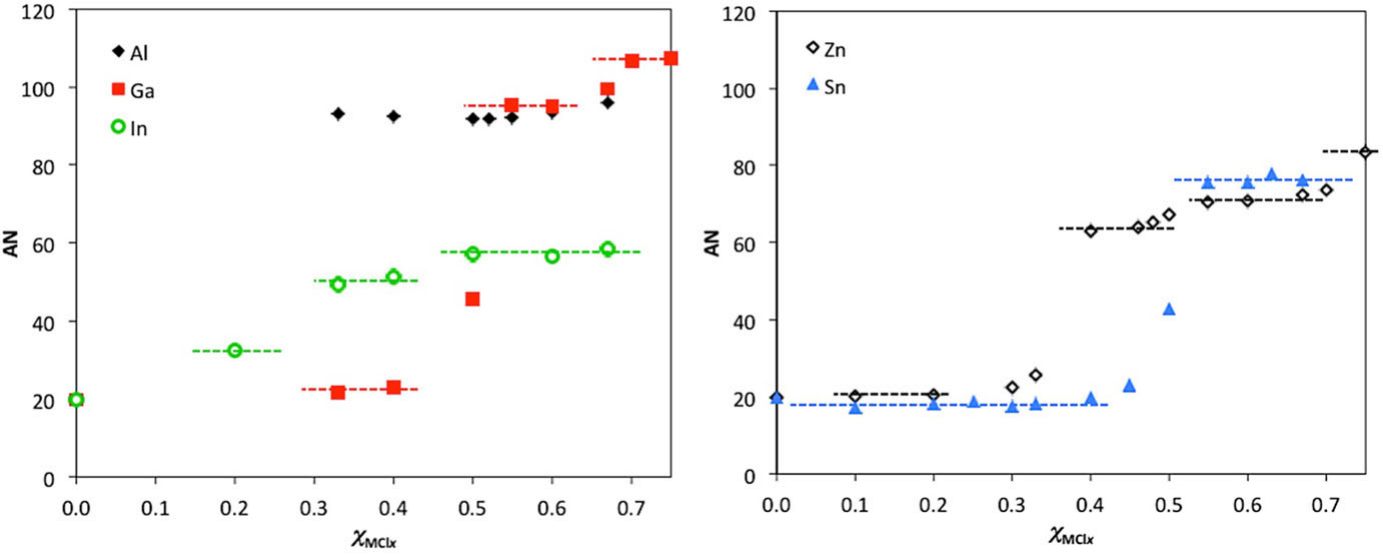
AN values for five [C_8_mim]Cl-MCl_*x*_ systems (M = Al, Ga, In, Zn and Sn^II^), plotted as a function of composition. Data adapted from [27, 58, 72]

In studying systems other than chloroaluminate(III), we found a very good agreement between AN values and speciation proposed for the given metallate [27, 58, 72]. To summarize these research efforts, AN values for five [C_8_mim]Cl–MCl_*x*_ systems (M = Al^III^, Ga^III^, In^III^, Zn^II^ and Sn^II^) are plotted, as a function of composition, in two graphs constituting Fig. 3. The [C_8_mim]Cl–AlCl_3_ system, in agreement with the literature, gave consistently high values across the compositional range. In [C_8_mim]Cl–GaCl_3_, AN = ca. 22 were recorded for basic compositions (*χ*_GaCl3_ < 0.50), with an increase to ca. 95 for acidic ones (*χ*_GaCl3_ < 0.50), and a further increase to AN = ca. 107 for *χ*_GaCl3_ = 0.67. These values can be assigned to consecutively dominant anions: [GaCl_4_]^−^, [Ga_2_Cl_7_]^−^ and [Ga_3_Cl_10_]^−^ (Table 2). Chloroindate(III) systems were reported to be mild Lewis acids, containing only monomeric anions: [InCl_6_]^3−^, [InCl_5_]^2−^ and [InCl_4_]^−^ (Eq. 5)—indeed, AN values increase stepwise to plateau at AN = ca. 57 for *χ*_InCl3_ ≥ 0.50 (Fig. 3). Chlorostannates(II) were reported to contain [SnCl_3_]^−^, equilibrated with [Sn_2_Cl_5_]^−^ at *χ*_SnCl2_ > 0.50 (Eqs. 9, 10), which is in agreement with one sharp change in Lewis acidity, from AN = ca. 18 at *χ*_SnCl2_ < 0.50 to AN = ca. 76 for *χ*_SnCl2_ > 0.50 (Fig. 3) [58]. In contrast, the [C_8_mim]Cl-ZnCl_2_ system was reported to contain multiple equilibrated anions, with Lewis acidic dimer, [Zn_2_Cl_6_]^2−^, occurring for *χ*_ZnCl2_ > 0.33—again, in agreement with the measured AN values (Fig. 3) [72]. For a handy reference, all measured AN values are listed in Table 3.

**Table 3:** AN values for five [C_8_mim]Cl-MCl_*x*_ systems (M = Al, Ga, In, Zn and Sn^II^), as a function of composition [27, 58, 72]

*χ*_MCl*x*_	AN values for [C_8_mim]Cl-MCl_*x*_, where M =				
	Al	Ga	In	Zn	Sn^II^
0.00	19.86	19.86	19.86	19.86	19.86
0.10				20.14	17.29
0.20			32.53	20.50	18.19
0.25					18.97
0.30				22.51	17.59
0.33	93.19	21.65	49.25	25.63	18.10
0.40	92.35	23.22	51.22	63.09	19.83
0.45					23.01
0.46				64.01	
0.48				65.24	
0.50	91.81	45.85	57.11	67.26	42.89
0.52	91.85				
0.55	92.19	95.30		70.55	75.45
0.60	93.30	95.09	56.53	70.86	75.66
0.63					77.91
0.67	95.95	99.54	58.36	72.49	76.14
0.70		106.71		73.57	
0.75		107.47		83.50	
1.00	85.60	75.90	74.30	66.00	75.20

From the comparison in Fig. 3, it transpires that chlorometallate ILs cover a wide range of Lewis acidities, from very mild to Lewis superacids (AN > 100), giving great promise for tunability in catalytic applications. At the same time, as already discussed, different probe molecules may give different orders of acidity [95], especially when acids with different nucleophilic centers are compared [96–98].

Aside from studying per se Lewis acidic ionic liquids, the AN approach was used to study donor/acceptor properties of non-halometallate ILs as solvents, in the spirit of Gutmann’s original work [24, 93, 99]. Although this is outside the scope of this review, it is worthy of note that AN values for such ionic liquids have been quantified using other spectroscopic techniques, such as Raman spectroscopy [100, 101], which could be of great use for studying halometallate ILs with paramagnetic nuclei.

Owing to their negligible vapor pressure, ILs give the unique opportunity of studying liquid matter via X-ray photoelectron spectroscopy (XPS), which is a high-vacuum technique. Licence and co-workers studied a range of chlorometallate systems as a function of their composition, and derived solvent properties from XPS spectra [25]. For chlorozincate(II) systems, a correlation between Zn 2p_3/2_ binding energies and AN values was observed, which opens up another strategy of quantifying Lewis acidity in ILs.

#### Adapted Solid-State Methods: FT-IR Spectroscopy

Lewis acidity in solid acids is commonly quantified employing vibrational spectroscopy and *N*-donor probes (pyridine, acetonitrile), typically used in near-stoichiometric quantities with respect to acidic sites. It is a very simple and time-efficient measurement, in which also paramagnetic ILs may be studied in contrast to NMR methods. A further advantage over the AN approach is the ability to distinguish between pyridine coordinating to Brønsted and Lewis acids, with vibrations for py-H and py-L.A. seen at ca. 1550 and 1450 cm^−1^ respectively [102]. Acetonitrile is sensitive only to Lewis acids, but not to Brønsted acids. A disadvantage, in the context of halometallate ionic liquids, is the need for high quantity of the probe (typically used at 0.33 mol ratio with respect to the metal center), which may disrupt equilibria in the liquid, as well as low sensitivity and resolution, compared to NMR spectroscopic probes.

In their seminal work, Kou et al. used both pyridine and acetonitrile as FT-IR spectroscopic probes to study Lewis acidity of a range of chlorometallate ILs [103]. The [C_4_mim]Cl–MCl_*x*_ series was studied, with Lewis acidity ranked as: Cu^I^ < Fe^III^ < Zn < Al, based on pyridine characteristic band vibration in acidic composition (Table 4). Studying the [C_4_mim]Cl-MCl_3_ system in several compositions, a py-L.A. band at 1448 cm^−1^ was assigned to [AlCl_4_]^−^, and 1454 cm^−1^ to [Al_2_Cl_7_]^−^. AcCN-L.A. band was detected for *χ*_AlCl3_ ≥ 0.55. A study on chlorozincate(II) ILs, within a broad compositional range (*χ*_ZnCl2_ = 0.25 − 0.75), revealed acidic band appearing for all *χ*_ZnCl2_ ≥ 0.33 compositions (Table 4), in agreement with Eq. 8 (but in contradiction to the speciation proposed in the same paper) [104]. Numerous papers followed afterwards, studying chlorometallates with different cations and varying combinations of metal chlorides and *χ*_MCl*x*_ values [105, 106]. Typically, a good agreement between the catalytic activity and py-L.A. vibrational frequency is reported, but—depending on the cation and the paper—the reports deviate ±2 cm^−1^ from exemplary data in Table 4, which indeed gives a very low resolution. In general, the authors using this approach are less concerned with physical and inorganic chemistry of the studied systems, but more focused on the catalytic performance of the studied ionic liquids.

**Table 4:** Py-L.A. vibrations chlorometallate ionic liquids, as a function of composition [103, 104, 107]

*χ*_MCl*x*_	Py-L.A. stretching frequencies (cm^−1^) for chlorometallate ILs, where *M* =					
	Al	Cu^I^	Fe	Zn	Sn^II^	Sn^II^
0.00	1438			1439		
0.33	1438, 1448			1439(sh), 1450		
0.40	1438, 1448					
0.50	1438, 1448			1439(sh), 1450		
0.55	1448					
0.60	1449, 1454(sh)					
0.67	1450(sh), 1454	1444	1446	1450	1449	1449
0.75				1450, 1454(sh)		

**Fig. 4:**
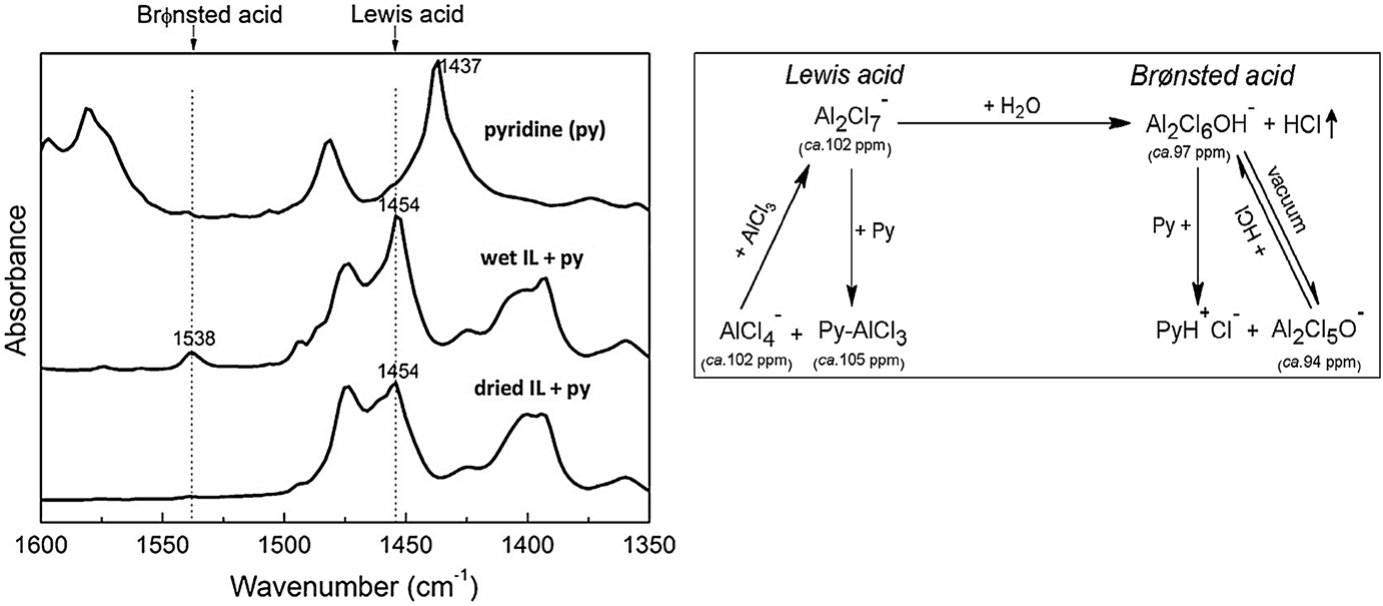
*Left* FT-IR spectra of neat pyridine compared to 10% pyridine in wet [HN_222_]Cl-AlCl_3_ (*χ*_AlCl3_ = 0.64), and 10% pyridine in high-vacuum dried [HN_222_]Cl-AlCl_3_ (*χ*_AlCl3_ = 0.64). *Right* Acidic species in the catalytic system, with assigned ^27^Al NMR chemical shifts. Adapted from [109]

Isobutene alkylation with 2-butene (refinery alkylation), catalyzed by chloroaluminate(III) ILs, is known to be enhanced by amount of CuCl and water (or protic additives). Several publications report on the use of pyridine as the IR probe to gain insight into Brønsted/Lewis acidity of this complex system [108–110]. In particular, Liu and co-workers combined ^27^Al NMR and IR spectroscopies to firstly identify Brønsted and Lewis acidic species in the system (Fig. 4) [109], and recently—to quantify them using pyridine titration method [110]. This distinction between Brønsted and Lewis species appears to be the key advantage of the IR spectroscopic approach.

#### Activity in Catalytic Process

In his seminal work, Olah and co-workers used Fridel–Crafts chemistry to measure the strength of Lewis acids [86]. Beckett et al. used epoxide polymerization rate, which correlated well with AN values measured by Gutmann–Beckett method for boron Lewis acids [111]. Hilt and Nödling used Diels–Alder reaction rates to rank Lewis acidity of silyl triflates, which correlated with the Δ*δ*(^2^H) values of deuterated quinolizidine-Lewis Acid adducts [112]. Finally, Kobayashi et al. studied both reaction rate and selectivity of the addition of a silyl enolate to an aldehyde and an aldimine, to form a complex classification of metal halides [87].

In ionic liquids, there were a number of publications where several Lewis acids were tested in a reaction, and ranked according to their strength [113]. Nevertheless, none of the above-cited standard reactions were adopted as the standard method. In the recent years, Diels–Alder has been gaining increasing popularity as the benchmark reaction of choice [75, 114–117].

### Selected Applications

Chlorometallate ionic liquids are used in a plethora of applications, not all of them directly related to their Lewis acidity (viz. electrochemistry). Here, three selected examples of applications of chlorometallate ILs as Lewis acids are presented: recent industrial applications, the development of supported ILs, and inorganic synthesis with Lewis acidic ionic liquids.

#### Refinery Alkylation with Chloroaluminate(III) Ionic Liquids

The review on industrial applications of ionic liquids by Seddon and Plechkova listed explicitly two industrial applications of Lewis acidic ILs: Difasol by IFP and Ionikylation by PetroChina—both using chloroaluminate(III) systems [118]. The interest of other major oil companies in this chemistry (BP, Exxon Mobil, Chevron) was inferred from a rich patent portfolio, owned by each company.

**Fig. 5:**
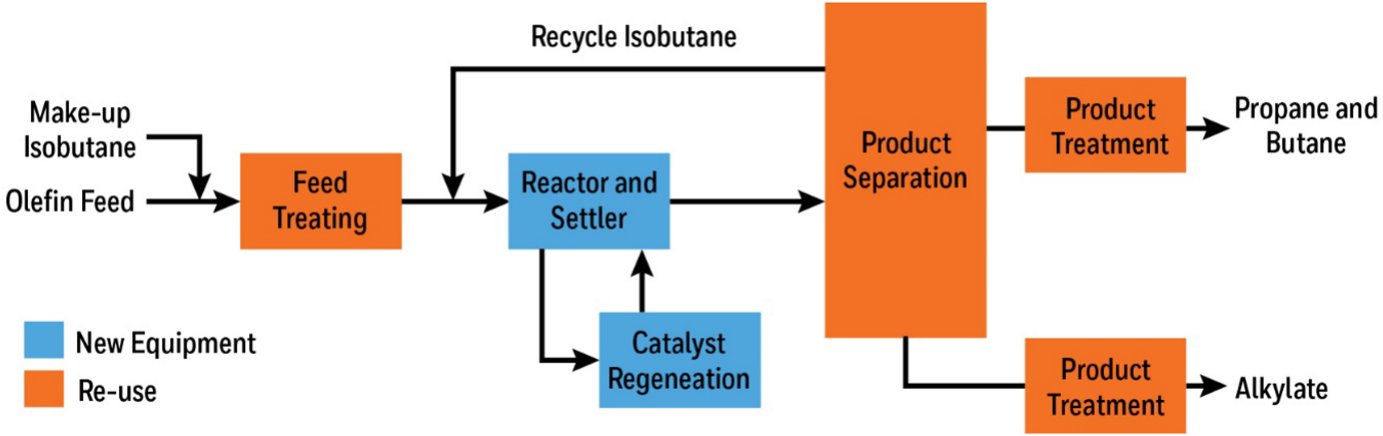
IONALKYLATION plant scheme showing retrofit strategy [125]

Currently, refinery alkylation catalyzed with chloroaluminate(III) ILs appears to be the center of attention of the many petrochemical companies. Firstly, in 2006 PetroChina reported retrofitting a 65 kt/y H_2_SO_4_ alkylation unit for use with a mixed chlorometallate (Al^III^/Cu^I^) ionic liquid in the Ionikylation process [119]. In 2013, a 100 kt/y plant was announced to operate successfully, a very similar catalytic system [composite ionic liquid alkylation (CILA)], with involvement from PetroChina, Shell, Deyang and National Science Foundation of China [120, 121]. In 2016 Chevron Phillips, who have been developing their ISOALKYL ionic liquid alkylation technology over the past 20 years, committed to converting a 4500 barrel per day HF alkylation unit with to the ISOALKYL technology, and has licensed the process through Honeywell UOP [122]. Plans to retrofit the alkylation unit (Fig. 5) in Utah, USA were to commence in 2017, pending planning permission [123]. This new technology set to reduce catalyst consumption and tackle the safety concerns associated with HF, in particular with its transport in bulk [124], as regeneration of the CIL catalyst can be performed on site. The ionic liquid process also has the advantage of being able to utilize more varied feedstocks than the liquid acid alkylation process and typically gives less conjunct polymers than liquid acid alkylations [125].

These industrial implementations of chloroaluminate(III) ILs in refinery alkylation have been underpinned by decades of research, some of them already highlighted in the discussion on quantifying acidity via FT-IR spectroscopy (Sect. 2.3.2). Refinery alkylates for use in high octane fuels had been produced on an industrial scale using either H_2_SO_4_ or HF as the acid catalyst [124], with aluminium(III) chloride described as too acidic and thus inducing side reactions, such as cracking [126]. However, Chauvin and co-workers reported the successful use of chloroaluminate ILs as catalysts for isobutane–butene alkylations, which was then expanded upon by Jess et al. [108, 127–129]. Tuneable acidity of the ionic liquid was found to play a crucial role: high *χ*_AlCl3_ values led to cracking (like neat AlCl_3_), whilst low acidity caused polyalkylations [130]. In addition to acidity control, it is very likely that charge stabilization of the transition-state charged intermediates, which occurs in ILs but not with AlCl_3_, contributes strongly to curtailing side reactions [131].

**Scheme 2:**
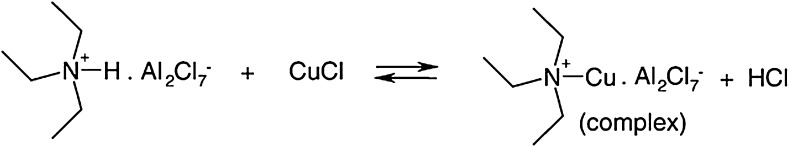
Proposed mechanism for the generation of a Brønsted acid from protic chloroaluminate (III) ionic liquid and copper (I) chloride [108]

Protic additives increased selectivity to the desired product up to a certain loading, but caused polyalkylation when that loading was exceeded (viz. speciation insight in Sect. 2.3.2) [128]. Addition of aromatic compounds also increased selectivity to the desired product, decreasing simultaneously light and heavy ends, which was attributed to an interaction between the Lewis acid and the π system of the aromatic ring [132]. Finally, studying metal halide additives, Liu and co-workers reported CuCl to increase the selectivity RON of the product [120, 133, 134]. Initially, it was attributed to the presence of a heterometallic chlorometallate anion, [AlCl_4_CuCl]^−^, which was suggested to be the origin of the peak at ca. 97 ppm in the ^27^Al NMR spectrum, and was observed in FAB-MS. However, other authors argued that the peak at ca. 97 ppm originates from a hydrolysis product, [Al_2_Cl_6_OH]^−^, which was confirmed by the drying of the sample under high vacuum, upon which the signal disappeared [109]. The same group proposed that Cu^I^ substitutes the proton in the [HN_222_]^+^ cation, generating HCl (Scheme 2). This also found spectroscopic confirmation in increased intensity of the py-H band in the FT-IR spectrum of CuCl-containing ionic liquid (viz. Sect. 2.3.2) [108].

Alkylation catalyzed with Lewis acidic ILs is currently of great interest, both commercially and scientifically, and more exciting new developments on both fronts can be expected.

#### SILP and SCILL

Homogenous and heterogenous catalysis both have their advantages and disadvantages, the former benefiting from good phase contact, no mass transfer limitation and control over catalytic species, and the latter from easy catalyst separation and low cost (with their disadvantages being the reverse) [135]. The strategy of depositing a thin layer of an ionic liquid on a solid support has been used to harvest benefits of both heterogenous and homogenous catalysis, with two core approaches being: supported ionic liquid phase (SILP) and more recently proposed solid catalyst with an ionic liquid layer (SCILL). The older SILP concept was shortly reviewed in 2006 [136], with broader reviews on both SILP and SCILL published in 2014 [137] and 2015 [138].

**Fig. 6:**
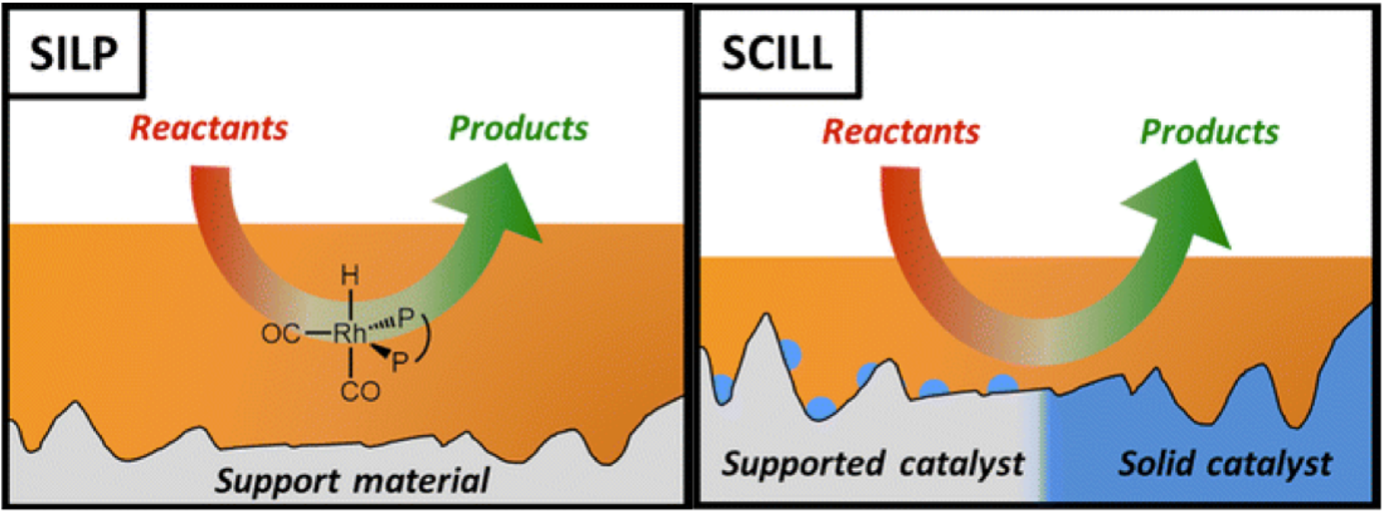
Pictorial representation of SILP and SCILL concepts. Modified from [138]

Both concepts utilize a typically highly porous support (silica, activated carbon, carbon nanotubes), which is coated with a small quantity of an ionic liquid, forming a thin layer. The IL may be either covalently tethered to the support material, usually by way of the cation, or simply adsorbed onto the surface. Covalently bound systems were reviewed in detail in 2016 [139]. In SILPs, the ionic liquid is either catalytically active, or has a catalyst dissolved in it in the form of coordination complex (Fig. 6, left). In SCILL, in contrast, the solid support is either catalytically active or has a solid catalyst deposited on the surface (viz. Pd/C), with the IL acting as a co-catalyst, stabilizing reactive species and/or altering the availability of reactants at catalytic sites (Fig. 6, right).

Initially, SILP systems did not utilize Lewis acidic ILs; notably, they are not even mentioned in the 2006 review on SILPs [136]. Wasserscheid and Haumann pioneered [C_2_mim][Al_2_Cl_7_]-containing SILPs as catalysts for the alkylation of cumene [140, 141]. The time required to reach 80% conversion was six times shorter with SILP, compared to the liquid–liquid biphasic reaction using the same ionic liquid. The catalyst could be recycled four times without the loss of activity. An increasing number of publications on chloroaluminate(III) SCILLs followed, predominantly testing known chemistry in the new engineering setting, and often reporting beneficial/synergistic effect of the support [138].

Chlorostannate(IV) ILs tethered to a silica support were used in Barbier–Prins condensation of paraformaldehyde and isobutene [142]. The SILP catalyst afforded the desired product (3-methy-3-buten-1-ol) with lower yield but higher selectivity compared to SnCl_4_, and in contrast to SnCl_4_—no leaching of tin was observed when SILP was used. Chlorostannate(II) and chlorozincate(II) ILs, supported on alumina, were used for gas sweetening (desulfurization) in the continuous mode [143]. Chrobok and co-workers used chloroaluminate(III) and chlorogallate(III) ILs, covalently tethered onto multimodal porous silica, in solventless Diels–Alder reaction [116]. Catalytic performance of Lewis acidic chloroaluminate(III) and chlorogallate(III) systems were equally good, but the chlorogallate(III) system was selected for the recycling studies due to higher moisture stability.

SCILL systems were introduced in 2012 by Wasserscheid and co-workers. The first example was the [C_4_mim]Cl–AlCl_3_ (*χ*_AlCl3_ = 0.67) ionic liquid on Pt/silica for the isomerization of *n*-octane in a slurry-phase reaction, under H_2_ pressure [144]. High selectivity was observed due to Pt centers catalyzing hydrogenation of undesired olefinic by-products, whereas enhanced reaction rate was attributed to increased Brønsted acidity in this unique Pt/H_2_/[C_4_mim]Cl–AlCl_3_ (*χ*_AlCl3_ = 0.67) system. Very recently, SCILLs with Lewis acidic ionic liquids were used for arene hydrogenation, and also in this case activity enhancement was observed [145], in analogy to earlier-reported homogenous study [131].

The study on SILP/SCILL materials with Lewis acidic systems is still in its infancy, and from the several papers published—it appears to be an interesting direction, opening up opportunities for ambitious fundamental studies on the nature of these complex catalytic systems.

#### Inorganic Synthesis

Ionic liquids in inorganic synthesis are typically used in a dual role of solvents and templating agents, often at relatively high temperatures (negligible boiling point results in low autogenic pressures). Developed initially by Morris and co-workers, this synthetic procedure is called ionothermal synthesis, in analogy to solvothermal synthesis in molecular solvents [146, 147]. Lewis acidic ILs, in particular haloaluminates, have been extensively used in ionothermal synthesis, the research direction led by Ruck and co-workers. In a recent review, the role of Lewis acidic systems in the synthesis of polycations of heavy main-group elements, such as Te, Bi, Sb–Se and Bi–Te, is extensively discussed [30]. Ionic liquids with Lewis acidic anions of a general formula [M_2_X_7_]^−^ act as solvents and scavengers for bases. Scavenging halides increases the solubility of certain precursors, e.g., enables dissociation of BiX_3_ to [BiX_2_]^+^ and X^−^, whereas scavenging water provides (to an extent) a self-drying reaction environment.

**Fig. 7:**
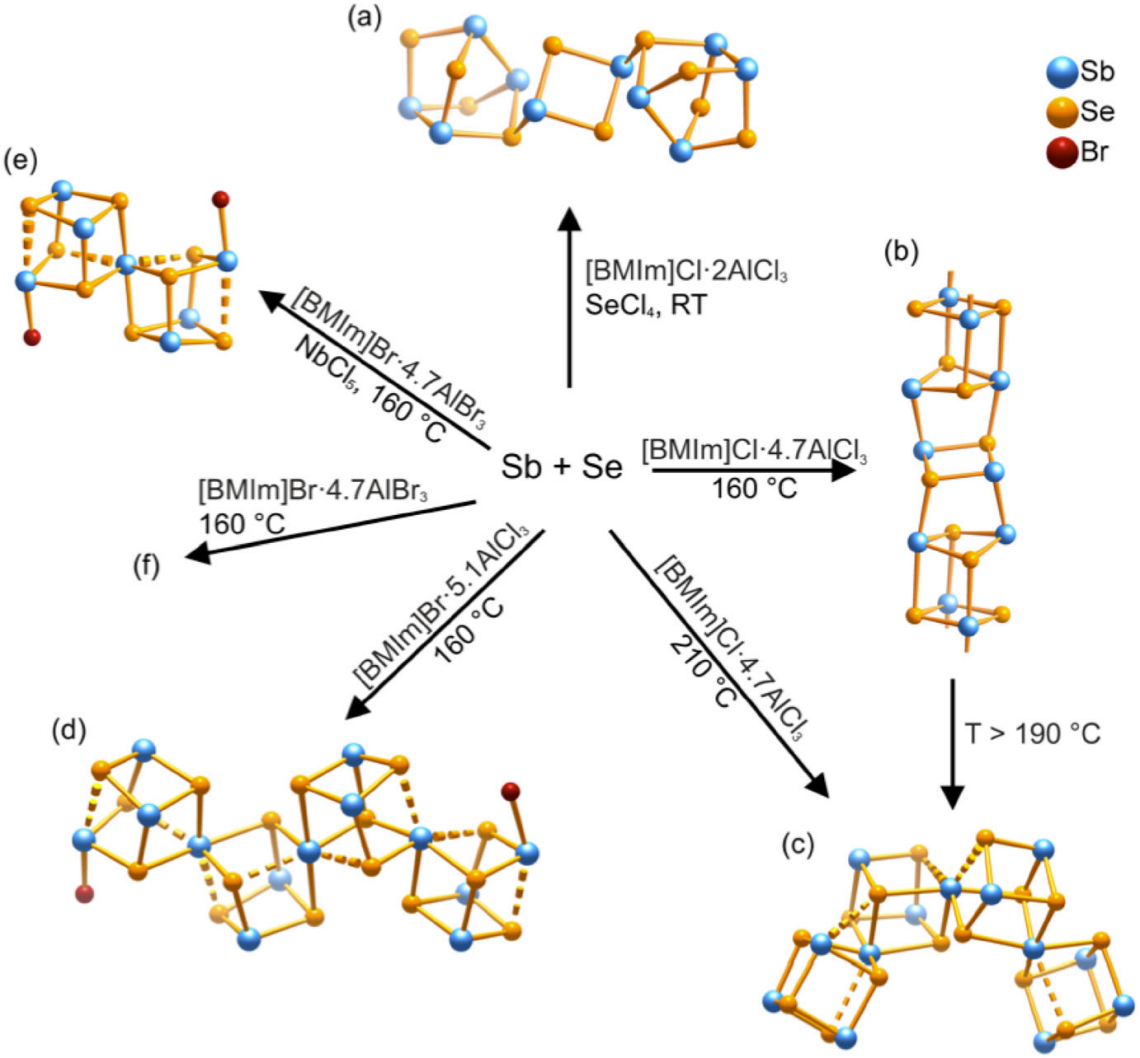
Structural diversity of antimony-selenium heteropolycations accessible in haloaluminate(III) ILs under various reaction conditions [30]

Altering the halide (Cl or Br) and the *χ*_AlX3_ value of haloaluminate(III) ILs, as well as the reaction temperature, was used to fine-tune the structure of the final product, as exemplified in Fig. 7 [30].

From the viewpoint of this review, particularly interesting is the manipulation of the composition (*χ*_AlCl3_) at different stages of synthesis. As shown in Fig. 7, typically a large excess of AlX_3_ is used during the reaction, above the formation of homogenous ionic liquid at room temperature (Fig. 1). This affords very weakly coordinating [Al_2_X_7_]^−^ anions, which are beneficial during the synthesis, but hinder the subsequent crystallization. This was addressed by Groh et al. by the addition of NaCl after the reaction, which shifts the equilibrium in Eq. 3 towards tetrahedral [AlX_4_]^−^, thus leading to crystallization [148]. The role of chloroaluminate(III) anions was studied computationally by Kirchner and co-workers [149]. A molecular precursor, Te_4_Br_2_, was studied in [C_2_mim]Cl and [C_2_mim]Cl-AlCl_3_, (*χ*_AlCl3_ = 0.57). Only in the latter system did dissociation of the precursor occur, proceeding through bromide abstraction by the Lewis acidic aluminium(III) center, yielding a cationic [Te_4_Br]^+^ species, and the [AlCl_3_Br]^−^ anion.

**Fig. 8:**
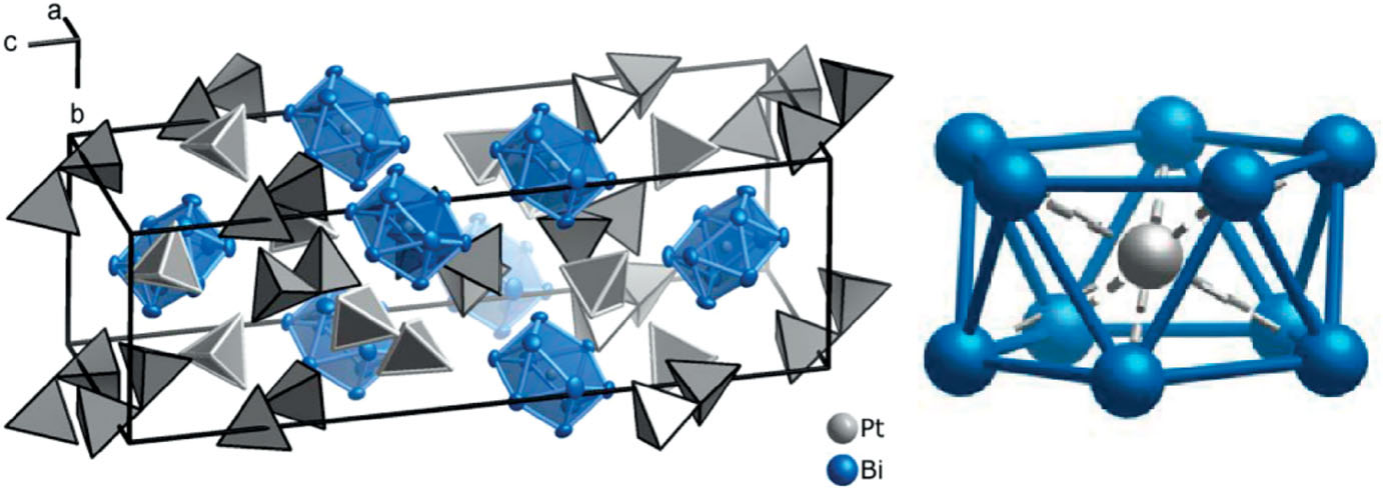
*Left* The crystal structure of [Pt@Bi_10_][AlBr_4_]_2_[Al_2_Br_7_]_2_, where *blue polyhedra* are the [Pt@Bi_10_]^4+^ cations, and *grey tetrahedra* are bromoaluminate anions: [AlBr_4_]^−^ with *white outlines*, [Al_2_Br_7_]^−^ with *black outlines*; *right* the structure of the *arachno* polycation, [Pt@Bi_10_]^4+^. The *ellipsoids* are at 90% probability level [153]

This area of inorganic chemistry is very prolific, and under constant development. The research started from accessing simple bismuth [150] and tellurium [151] polycations, developed into the synthesis of cluster compounds, such as Sn^II^[Sn^II^Cl][W_3_Cl_13_] [152], to recently reported clusters with metal inclusions, such as [Pt@Bi_10_][AlBr_4_]_2_[Al_2_Br_7_]_2_ (Fig. 8) or [Pd@Bi_10_][AlBr_4_] [153]. It is apparent that a plethora of synthetic opportunities await here, possibly using non-haloaluminate(III) ILs to open up another dimension of study.

## Liquid Coordination Complexes (LCCs)

### Historical Context

Combinations of a metal halide, in most cases is aluminium(III) chloride, and an aprotic organic donor, such as THF, acetonitrile or dimethylformamide, are used as Lewis acidic reagents in organic chemistry. The Lewis basic donor is used to modify the Lewis acidity/reactivity of the metal halide [154–156]. Typically, reactions are performed in solutions, and ratios of metal halide to donor vary in a wide range. Speciation and physical properties of neat reagents are never the focus of such studies, but in isolated cases they have been mentioned to be liquids rather than solids.

In coordination chemistry, adducts of aluminium halides with organic donor molecules have been demonstrated to crystallize as either neutral molecular complexes, such as [AlCl_3_(THF)] and [AlCl_3_(THF)_2_], or ionic species, e.g., [Al_2_Cl_2_(THF)_4_][AlCl_4_]—with symmetric or asymmetric splitting of the ‘Al_2_Cl_6_’ unit taking place depending on the concentration and reaction conditions [157, 158].

Experience with halometallate ILs allowed for observation of these low melting metal–ligand combinations in a new light, and rather than seeing an ‘oil’ that failed to crystallize, an opportunity to develop a new generation of Lewis acidic liquids was spotted. Herein, such materials are referred to as liquid coordination complexes (LCCs). Much like halometallate ILs evolved from molten salts, LCCs were developed as a less expensive and easier to synthesize alternative to chlorometallate ionic liquid [159, 160].

### Speciation

**Fig. 9:**
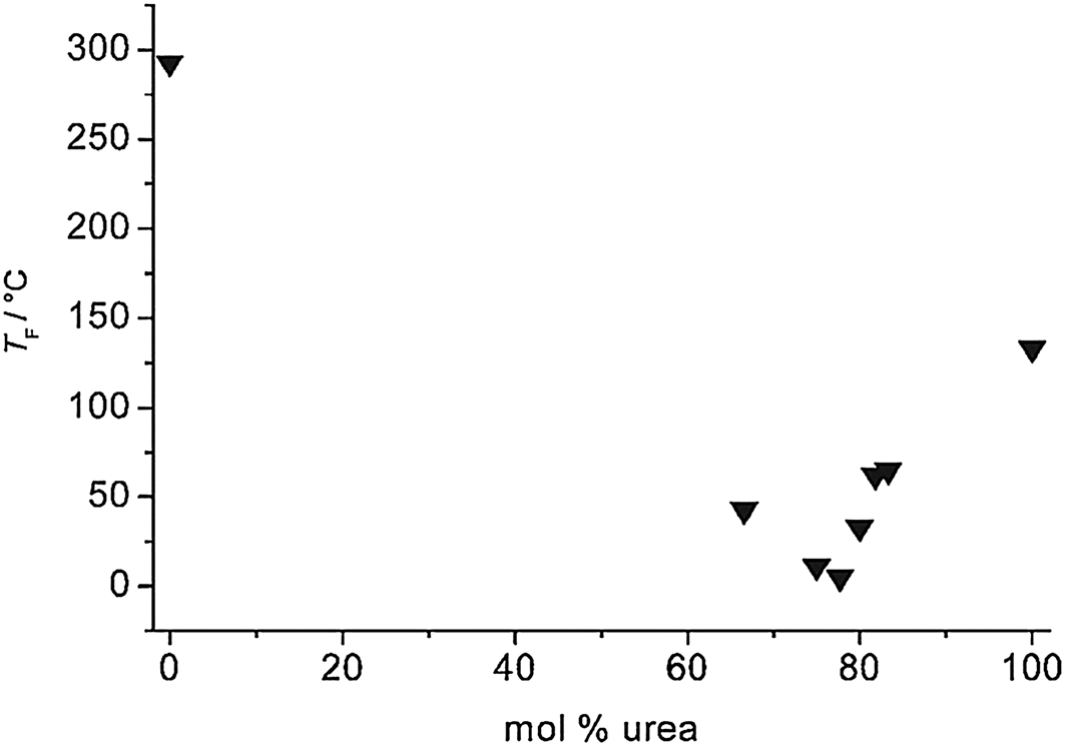
Phase diagram urea–ZnCl_2_ system as a function of composition [159]

In 2007, Abbott and co-workers reported that urea formed ambient-temperature eutectics with SnCl_2_ and FeCl_3_ [159]. ZnCl_2_ was reported to form eutectics with urea (at eutectic point *χ*_ZnCl2_ = 0.22, or 78% urea—see Fig. 9), acetamide (*χ*_ZnCl2_ = 0.20), ethylene glycol (*χ*_ZnCl2_ = 0.20) and hexanediol (*χ*_ZnCl2_ = 0.25). The Lewis acidity of these liquids has never been studied, but it is unlikely for them to be of significant Lewis acidity, considering the excess of basic urea (ca. 4:1).

Subsequently, eutectic mixtures of AlCl_3_ and urea, acetamide or *N*,*N*-dimethylurea were reported (0.50 ≤ *χ*_AlCl3_ ≤ 0.60) [161]. Swadźba-Kwaśny and co-workers described a range of L-MCl_3_ combinations, where M = Al or Ga, and L = acetamide (AcA), urea (Ur), thiourea (SUr), trioctylphosphine (P_888_) or trioctylphosphine oxide (P_888_O), with varying metal chloride proportions (0.50 ≤ *χ*_AlCl3_ ≤ 0.60 and 0.50 ≤ *χ*_GaCl3_ ≤ 0.75) [160]. Liu and co-workers studied combinations of AlCl_3_ with acetamide, *N*-methylacetamide and *N,N*-dimethylacetamide (0.51 ≤ *χ*_AlCl3_ ≤ 0.60) [162]. Finally, Dai and co-workers reported on mixtures of AlCl_3_ and 4-propylpyridne (0.52 ≤ *χ*_AlCl3_ ≤ 0.60) [163] and dipropylsulphide (up to *χ*_AlCl3_ = 0.51) [164].

**Fig. 10:**
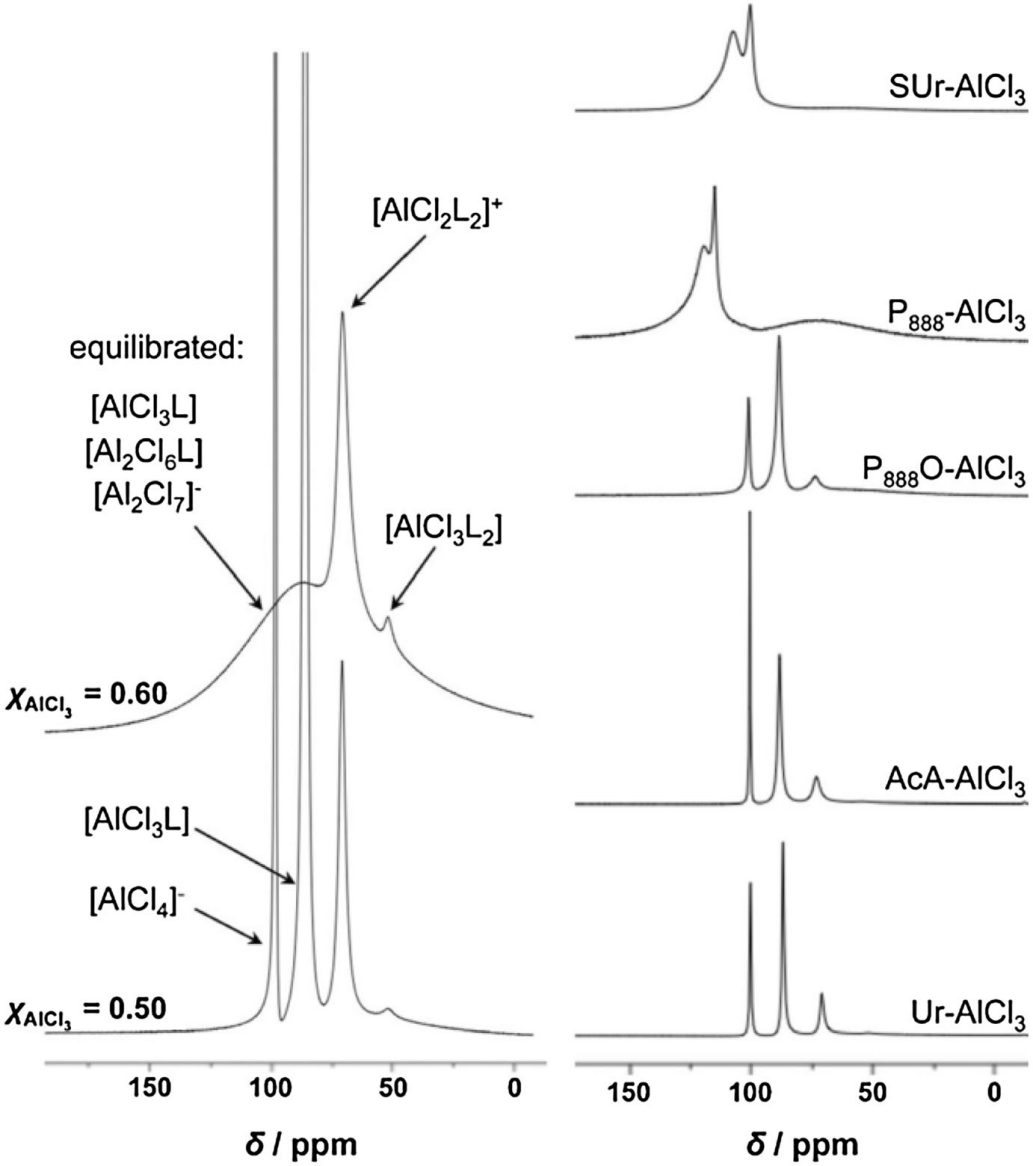
^27^Al NMR spectra of AcA-AlCl_3_ *χ*_AlCl3_ = 0.50 and 0.60 (*left*), and L-AlCl_3_ *χ*_AlCl3_ = 0.50 (*right*) [160]

Speciation studies on AlCl_3_ and GaCl_3_ combined with above-listed *O*-, *P*- and *S*-donors, based on ^27^Al NMR and Raman spectroscopy, revealed equilibrated neutral, cationic and anionic complexes of aluminium (Fig. 10) [160].

Naturally, speciation of LCCs as a function of *χ*_MCl3_ was different from that determined for chloroaluminate(III) and chlorogallate(III) ILs (Table 2). For *χ*_MCl3_ = 0.50, all mixtures contained only monomeric complexes: [MCl_2_L_2_]^+^, [MCl_3_L] and [MCl_4_]^−^ (Eq. 14), whereas at *χ*_MCl3_ = 0.60, there were dimeric, Lewis acidic species prevalent: [M_2_Cl_6_L] and [M_2_Cl_7_]^−^, in addition to mononuclear cation, [MCl_2_L_2_]^+^ (Eq. 15).

14 $$2{\text{L}} + 2{\text{MCl}}_{3} \to 2\left[ {{\text{MCl}}_{3} {\text{L}}} \right] \rightleftarrows \left[ {{\text{MCl}}_{2} {\text{L}}_{2} } \right]\left[ {{\text{MCl}}_{4} } \right]$$

15 $$2{\text{L }} + \, 3{\text{MCl}}_{3} \to \left[ {{\text{MCl}}_{3} {\text{L}}} \right] \, + \, \left[ {{\text{M}}_{2} {\text{Cl}}_{6} {\text{L}}} \right] \rightleftarrows \left[ {{\text{MCl}}_{2} {\text{L}}_{2} } \right]\left[ {{\text{M}}_{2} {\text{Cl}}_{7} } \right]$$

For higher metal chloride loadings (*χ*_MCl3_ > 0.60), AlCl_3_ was found to precipitate, whereas GaCl_3_-LCCs remained liquid until *χ*_MCl3_ = 0.75, suggesting the formation of oligomeric, highly Lewis acidic complexes (Eq. 16) [160]. This is in agreement with findings for chlorometallate ILs, where only mono- and dinuclear chloroaluminate(III) anions are stable, but chlorogallate(III) anions have the ability to form higher oligomers (viz. Table 2).

16 $$2{\text{L}} + 4{\text{MCl}}_{3} \to 2\left[ {{\text{Ga}}_{2} {\text{Cl}}_{6} {\text{L}}} \right] \rightleftarrows \left[ {{\text{GaCl}}_{2} {\text{L}}_{2} } \right]\left[ {{\text{Ga}}_{3} {\text{Cl}}_{10} } \right]$$

Further refinement upon this speciation was proposed by Liu and co-workers, who studied the amide-AlCl_3_ combinations [162]. All three ligands: AcA, NMA and DMA have the potential to act as *O*-donors and *N*-donors. It has been demonstrated that acetamide coordinated exclusively through the oxygen atom, with a small contribution from Al–N coordination mode in NMA, and a larger Al–N contribution in DMA. LCCs containing methylated acetamides had a higher proportion of [AlCl_4_]^−^ than acetamide in the same composition, which the authors interpreted as the formation of [AlCl_2_(η^2^-L)]^+^ in the former case, shifting equilibrium in Eq. 16 to the right.

**Fig. 11:**
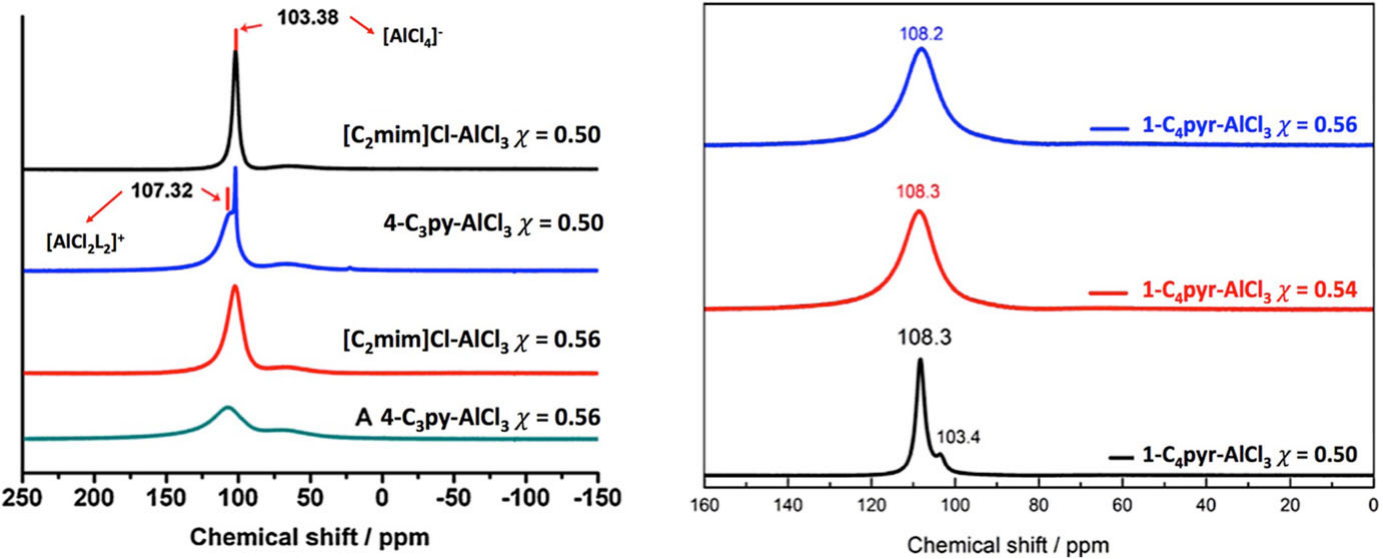
Comparison of ^27^Al NMR spectra. *Left* ionic liquid, [C_2_mim]Cl-AlCl_3_, and an LCC, 4-C_3_py-AlCl_3_, at two molar ratios of aluminium(III) chloride: *χ*_AlCl3_ = 0.50 and 0.56. *Right* 1-C_4_py-AlCl_3_, at three molar ratios of aluminium(III) chloride: *χ*_AlCl3_ = 0.50, 0.54 and 0.56. *Left* Adapted from [163]. *Right* Adapted from [165]

Mixtures of 4-propylpyridne and AlCl_3_, liquid at 0.52 ≤ *χ*_AlCl3_ ≤ 0.60, were investigated using a combination of multiple techniques: ^27^Al NMR and FT-IR spectroscopy, mass spectrometry, TGA, DSC and viscometry, followed by comprehensive electrochemical study [163]. ^27^Al NMR spectra were compared to these of an archetypical chloroaluminate(III) IL, [C_2_mim]Cl-AlCl_3_ (Fig. 11).

The existence of an ionic compound, [AlCl_2_(4-proylpyridine)_2_][AlCl_4_], at *χ*_AlCl3_ = 0.50, is postulated based on ^27^Al NMR spectroscopy and mass spectrometry. For *χ*_AlCl3_ = 0.52, no [Al_2_Cl_7_]^−^ was observed in mass spectra—in accordance to earlier reports of Abbott and co-workers [161]. This might be disputed due to problems with using mass spectrometry for speciation of chlorometallate ILs in general [14, 72]. However, the conclusion is supported by Endres and co-workers, who studied a similar system, 1-butylpyrolidine and AlCl_3_ (liquid for 0.47 ≤ *χ*_AlCl3_ ≤ 0.55), using more reliable in this context vibrational spectroscopy, and also have seen no evidence for the Lewis acidic [Al_2_Cl_7_]^−^ anion [165].

Based on very interesting changes in conductivity found from their electrochemical studies, Dai and co-workers suggest the presence of equilibria found in Eqs. 17 and 18 [163].

17 $$2{\text{AlCl}}_{3} \rightleftarrows \left[ {{\text{AlCl}}_{2} } \right]^{ + } + \, \left[ {{\text{AlCl}}_{4} } \right]^{ - }$$

18 $$\left[ {{\text{AlCl}}_{2} } \right]^{ + } + \left[ {{\text{AlCl}}_{2} \left( {4 - {\text{C}}_{3} {\text{py}}} \right)_{2} } \right]^{ + } \rightleftarrows \, 2\left[ {{\text{AlCl}}_{2} \left( {4 - {\text{C}}_{3} {\text{py}}} \right)} \right]^{ + }$$

Should such species exist, this liquid should be extremely Lewis acidic due to the presence of a dicoordinate cation, [AlCl_2_]^+^. However, such cation has never been reported in the literature, and there is no direct, spectroscopic evidence for its existence presented in the paper. Chemical shifts in ^27^Al NMR spectroscopy depend strongly on coordination number and geometry around the studied nuclei. In general, tetracoordinate complexes of Al(III) give signals around 100 ppm, pentacoordinate around 50 pm and hexacoordinate around 0 ppm [166]. ^27^Al NMR spectra of the 4-C_3_py-AlCl_3_ system feature two signals at 103 and 107 ppm, assigned to tetracoordinate [AlCl_4_]^−^ and [AlCl_2_(4-C_3_py)_2_]^+^, respectively. The same range of chemical shifts is recorded by Endres and co-workers [165]. There was no indication of an extremely deshielded ^27^Al NMR signal that would suggest the presence of the previously unreported [AlCl_2_]^+^.

### Acidity Measurements

There is relatively little information published with regards to quantified Lewis acidity of LCCs. Coleman et al. reported AN = 103 for DMA-GaCl_3_, *χ*_GaCl3_ = 0.60, and AN = 96–103 (several signals for the probe were recorded) for DMA-AlCl_3_, *χ*_AlCl3_ = 0.60. These are slightly higher than acceptor numbers recorded for the corresponding chlorometallate ILs (AN = 93–95) [27]. Yet unpublished data recorded by our group indicate that, in many cases, multiple signals are recorded for TEPO dissolved in LCCs, therefore it requires a careful consideration how these could be presented in a meaningful manner.

### Applications

In analogy to halometallate ionic liquids, there are two major strands emerging in the newly developing LCC research: electrochemistry (led by Abbott, Dai, Endres) and Lewis acidic catalysis (Swadźba-Kwaśny, Liu). The focus of this review is on the latter strand of work.

The first catalytic studies using LCCs were on carbocationic reactions. Abbott reported the acetylation of ferrocene with acetic anhydride, promoted by Ur–AlCl_3_ and AcA–AlCl_3_ (both *χ*_AlCl3_ = 0.60), which led to a mixture of mono- and diacetylated products [161]. Interestingly, both systems gave significantly different results, with markedly higher conversion to diacetlyated product for AcA–AlCl_3_ (both *χ*_AlCl3_ = 0.60), which indicates higher Lewis acidity of the later.

**Fig. 12:**
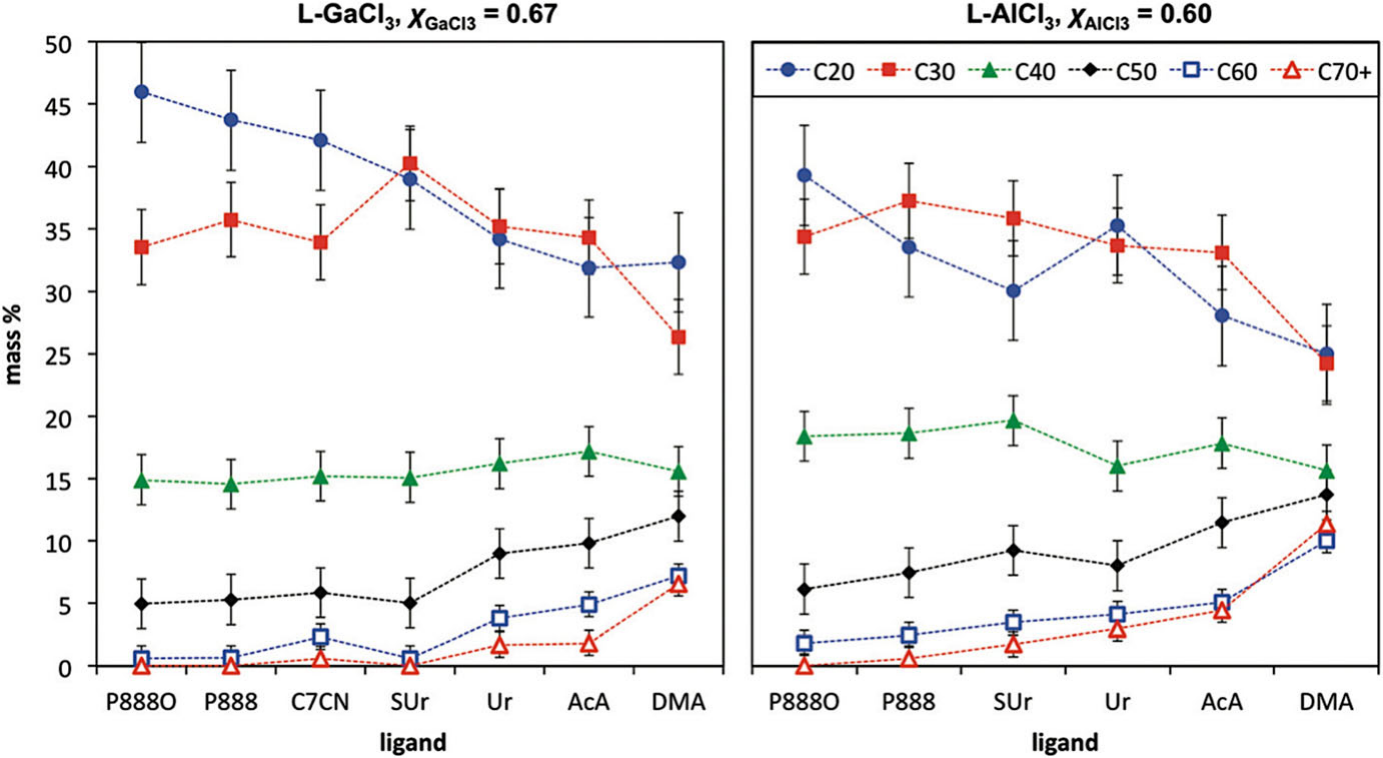
Distributions of 1-decene oligomers, produced in l-MCl_3_-catalyzed oligomerizations (120 °C, 1.707 mmol of LCC), where M = Al or Ga, and L = P888O, P888, C7CN (octanenitrile), SUr, Ur, AcA or DMA [167]

Swadźba-Kwaśny and co-workers used a range of LCCs based on AlCl_3_ and GaCl_3_ for the oligomerization of 1-decene to polyalphaolefins (PAOs) [167]. Low-viscosity polyalphaolefins (PAO_4_ and PAO_6_ lubricant base oil grades) were obtained, with all quantified physical parameters aligned with the industrial requirements for the commercial products. Changing metal and ligand, distribution of oligomers could be tuned within a wide range (Fig. 12). Particularly significant was the stark contrast between distribution of oligomers achieved with acetamide (AcA), and structurally very similar dimethylacetamide (DMA), which highlights the influence of speciation of LCCs on their catalytic performance (viz. research of Liu and co-workers, discussed in Sect. 3.2) [162].

l-GaCl_3_ LCCs were used to catalyze Friedel–Crafts alkylation of benzene with 1-decene [168]. Although higher reaction rates were achieved with L = Ur or DMA, lipophilic ligands (L = P_888_ or P_888_O) enabled easy phase separation from the product. Working at lower conversions (ca. 50%) gave very high selectivities to the desired 2-phenyldecane (up to 50%).

Liu and co-workers used amide-AlCl_3_ systems to catalyze refinery alkylation of isobutene with 2-butene to high-octane fuels [169]. Compared to chloroaluminate(III) ILs, LCCs gave less polyalkylates and better selectivity, at the same composition (*χ*_AlCl3_ = 0.60). In analogy to the PetroChina process, the addition of CuCl increased RON, up to a maximum value of RON 98.40.

Finally, an LCC, Ur-AlCl_3_ *χ*_AlCl3_ = 0.60, and a chloroaluminate(III) ionic liquid, [C_4_mim]Cl-AlCl_3_ *χ*_AlCl3_ = 0.67, were supported on Pd/C to form SCILL, and used as co-catalysts for the hydrogenation of toluene [145]. Both catalysts saw an increased rate of hydrogenation compared to the uncoated catalyst. Interestingly, at lower temperatures (<60 °C) the IL was a more effective co-catalyst, whereas at elevated temperatures (>60 °C) the LCCs co-catalyst performed better. This was attributed to significant changes in speciation of LCC as a function of temperature, already demonstrated by variable temperature ^27^Al NMR spectroscopy [160, 162].

## Ionic Liquids with Lewis Acidic Cations

Lewis acidic species are characterized by the local electron density deficiency. Lewis acidity is therefore enhanced by the positive change on the Lewis acidic center. This has been exploited in main-group chemistry, with increasing interest in borocation, phosphorus cations, and other positively charged species as strong Lewis acids. It is therefore ironic that, in the field of ILs (which lends itself to manipulation of ionic species), the overwhelming majority of Lewis acids are Lewis acidic at the anion! Only very recently, a handful of ILs bearing Lewis acidic cations were reported, but the field is already expected to grow rapidly. Structurally, these newly reported examples can be divided into two categories: solvate ionic liquids and borenium ionic liquids.

### Solvate Ionic Liquids

#### Historical Context

**Fig. 13:**
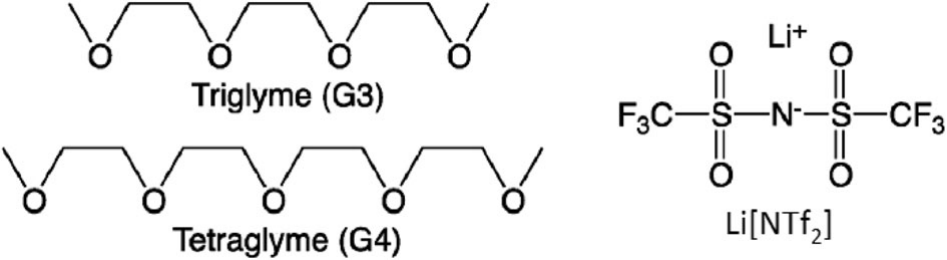
The structure of components of two solvate ionic liquids: [Li(G3)][NTf_2_] and [Li(G4)][NTf_2_] [172]

It has been demonstrated that certain complexes of lithium have very low melting points; for example, whereas [Li(G1)][NTf_2_] and [Li(G2)][NTf_2_] are solids, [Li(G3)][NTf_2_] is liquid at room temperature [170]. In 2010, Watanabe and co-workers reported liquid complexes of glymes (G*n*) and lithium salts, designed in analogy to crown ether complexes of alkali metals, to be ionic liquids [171]. Equimolar mixtures of Li[NTf_2_] and triglyme, G3, or tetraglyme, G4 (Fig. 13), formed liquid materials of high thermal stability, high conductivity (0.6–1.6 mS cm^−1^ at 30 °C) and low viscosity (68.0–156.0 mPa s) [171].

Following Angell’s categorization of ionic liquids, [12] these were classified as solvate ILs, because the cation was solvated by a neutral glyme [173]. In subsequent years, the studies were expanded, predominantly by Watanabe and co-workers, to include Group 1 and 2 metal salts [174], with various non-coordinating anions, primarily [NTf_2_]^−^, [OTf]^−^ and [BETI]^−^ [175], and a range of glymes [176]—all with the outlook to produce electrolytes for metal-ion batteries. Kitada et al. studied the electrochemistry of concentrated solutions of AlCl_3_ in glymes, which were shown to contain [AlG*n*]^3+^ cations and [AlCl_4_]^−^ anions [177, 178]. Just recently, the Lewis acidity of solvate ILs was investigated, with only lithium cations covered to date [114, 179].

#### Speciation

Like other families of Lewis acidic liquids discussed in this work, solvate ILs are characterized by complex dynamic equilibria. The main one is the competition between the cation–anion interaction and the ligand–cation interaction (Eq. 19) [175].

19 $${\text{M}}\left[ {\text{A}} \right] + {\text{Gn}} \rightleftarrows \left[ {{\text{M}}\left( {\text{Gn}} \right)} \right]\left[ {\text{A}} \right]$$

If the equilibrium is shifted to the left, the liquid behaves like a solution of a salt in molecular solvent; upon shifting to the right, it has properties of ionic liquid. In IL, the glyme is coordinated in a multi-dentate mode to the metal center, and therefore: (1) has negligible or significantly lowered vapor pressure [173], (2) is not corrosive to polysulfide electrodes, in contrast to free glyme [180]. Basic anions are more likely to closely associate with the alkali metal center, thus causing the release of free glyme, whereas [NTf_2_]^−^, [ONf]^−^ and [PF_6_]^−^ generate ILs [181]. In addition, a good cavity size match is important: Li^+^ matches with G3 or G4, Na^+^ with G4 and G5 and K^+^ with G5 [174, 181, 182]. Ligands with lower hapticity, such as THF or G2 do form [Li(THF)_4_]^+^ or [Li(G2)_2_]^+^, but overall Li^+^-ligand binding energy is obviously weaker, thus concentrated electrolyte solutions are formed [176].

**Fig. 14:**
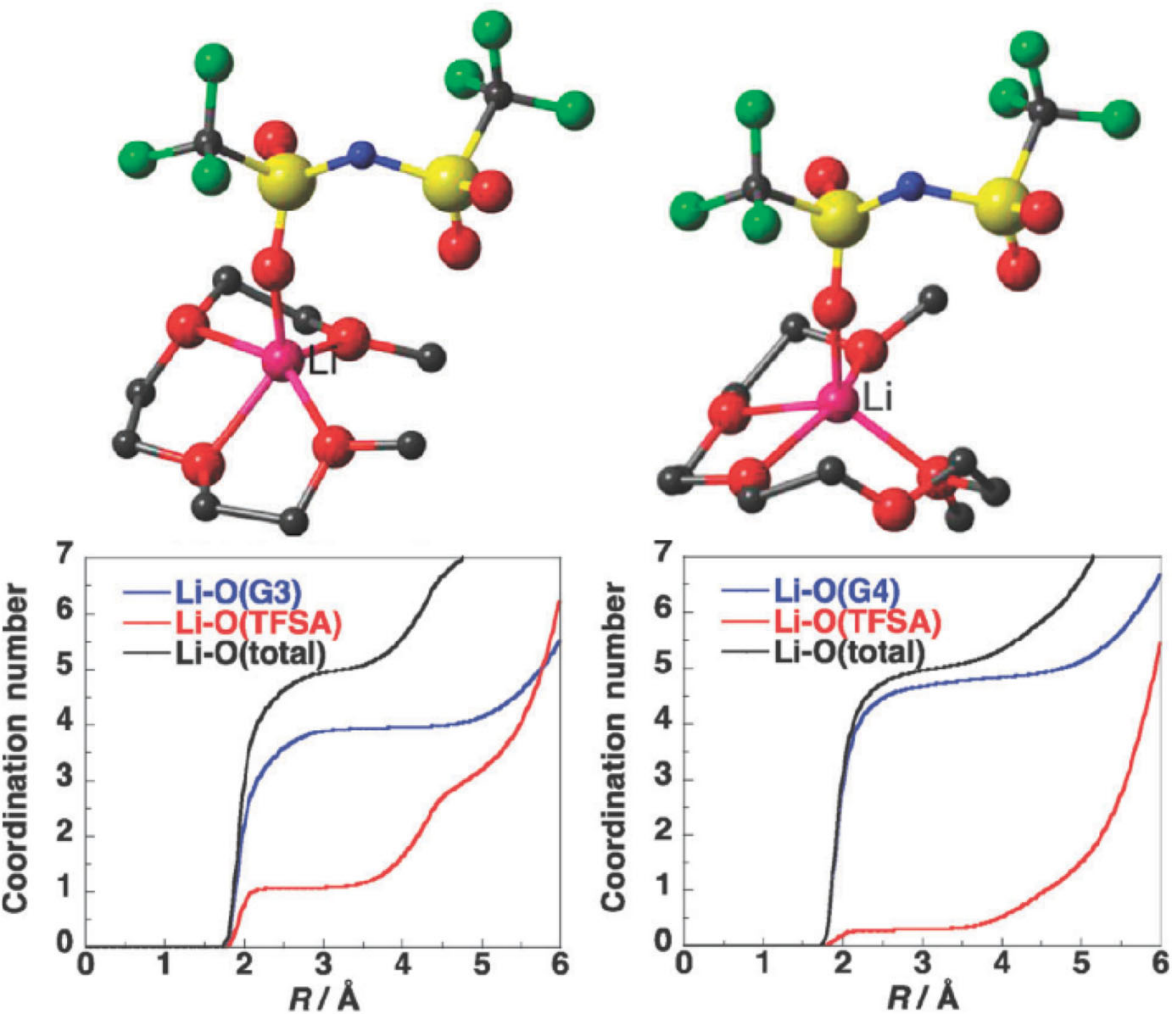
Optimized structures and coordination numbers of [Li(G3)][NTf_2_] (*left*) and [Li(G4)][NTf_2_] (*right*). Structures: Li *purple*, O *red*, C *silver*, N *blue*, S *yellow*, F *light blue* (H atoms omitted for clarity) [172, 173]

Details of coordination depend on metal–glyme–anion combination. For example, molecular dynamics simulations of equimolar mixtures of G3 or G4 with Li[NTf_2_] revealed that the first shell of lithium in [Li(G3)][NTf_2_] contained 4 oxygen atoms from G3 and 1 oxygen from the sulfonyl group of the anion, whereas the first shell of lithium in [Li(G4)][NTf_2_] contained 4.5 oxygens from G4, and 0.5 oxygen from [NTf_2_]^−^ (Fig. 14) [172].

#### Acidity Measurements

In contrast to earlier-discussed ILs, Lewis acidity of solvate ILs has been probed by Kamlet–Taft parameters. The Kamlet–Taft method uses solvachromatic dyes to determine three independent parameters of a liquid polarity: hydrogen bond donor ability (α), hydrogen bond acceptor ability (*β*), and dipolarity/polarizability (π*)—the last one interpreted as the sum of all interactive forces between molecules which do not result in a chemical change [183]. Kamlet–Taft measurements arise from changes to spectroscopic shifts (UV/Vis) from a solvochromatic probe molecule dissolved in the solvent. The approach, well-established for molecular liquids for which it accurately describes solvent polarizability parameters, has been popularly used also in ionic liquids. Holzweber, Linert and co-workers challenged the validity of the Kamlet-Taft in ILs, arguing that cations and anions affect each other donor/acceptor abilities, and thus interaction with the dyes, possibly rendering the numbers meaningless [99]. However, with the aid of computational chemistry it is viable to elucidate the contribution of each ionic liquid component on the *α*, *β* and π* values [179]. *N.B*., strongly Lewis acidic ILs, e.g., chloroaluminate(III) systems, can promote decomposition of the dye, preventing meaningful UV–Vis measurements [184].

Kamlet–Taft parameters are not typically employed to report on Lewis acidity; rather, the α parameter is associated with hydrogen bond donor strength/Brønsted acidity. However, Henderson and co-workers demonstrated that, in the absence of acidic protons, *α* can be interpreted as the measure of Lewis acidity [179]. Moreover, in their original paper, Kamlet and Taft introduced a variety of equations for comparison of their data to allow conversion between the π*, *β* and α parameters and the Gutmann acceptor number (see Sect. 2.3.1), allowing for specific data comparisons [183].

The values of *α*, *β* and π* parameters, measured by Henderson and co-workers for [Li(G3)][NTf_2_], [Li(G4)][NTf_2_] and the glymes, are listed along polarity and AN values (Table 5) [179]. The *α* parameters for solvate ILs were much higher than the value recorded for [C_4_mim][NTf_2_], 1.3 vs. 0.6. In the absence of strong hydrogen bond donors, this high value was attributed to Lewis acidity of the lithium center, which was supported by a computational model. It was suggested that the interaction of the probe (a model base) with Li^+^ occurs by replacing the weakly coordinated anion (viz. Fig. 14) in the axial position.

**Table 5:** Polarity (*E*_N_^T^), Kamlet–Taft parameters (*α*, *β* and π*) and acceptor numbers (AN) for solvate ILs: [Li(G3)][NTf_2_] and [Li(G4)][NTf_2_], compared to neat glymes, G3 and G4, and a benchmark IL, [C_4_mim][NTf_2_]. Adapted from [114, 179, 185, 186]

Solvent	*E*_T_^N^	*α*	*β*	π*	AN
[Li(G3)][NTf_2_]	1.03	1.32	0.41	0.94	26.5
[Li(G4)][NTf_2_]	1.03	1.35	0.37	0.90	26.5
G3	0.30	0.01	0.96	0.65	0.23
G4	0.28	0.05	0.96	0.67	0.23
[C_4_mim][NTf_2_]	0.59	0.59	0.29	0.96	11.9
[C_4_mim][NTf_2_]	0.55	0.61	0.24	0.98	3.10

**Fig. 15:**
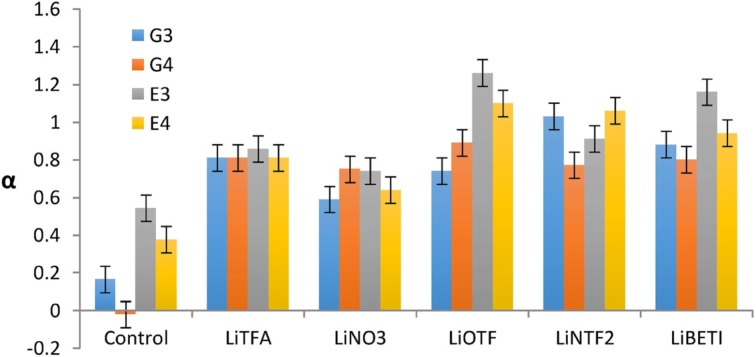
The *α* values of equimolar mixtures of Li[A] salts and glymes (G*n*) or glycols (E*n*). The control values are pure G*n* or E*n* [187]

Almost in parallel, Warr and co-workers published an extensive study on Kamlet–Taft parameters for a wide range of solvate ILs [187]. Corroborating with Henderson and co-workers, they note striking increase in *α* values for solvate ILs, compared to pure glymes. Moreover, higher *α* values are obtained for ‘good ionic liquids’ with weakly coordinating anions, than for ‘poor ILs’ with more basic anions (Fig. 15)—this corroborates with Henderson’s postulate that the base replaces/competes with the anion in coordinating to the lithium center, and possibly points towards future direction in design of Lewis acidic ILs with alkali metal cations.

Henderson and co-workers measured also acceptor numbers for [Li(G3)][NTf_2_] and [Li(G4)][NTf_2_] [114, 179]. AN values were estimated at AN = 26.5 for both systems, compared to AN = ca. 0.2 for the glymes, and AN = 11.9 and 3.10 (two signals recorded) for a benchmark ionic liquid, [C_4_mim][NTf_2_] [114]. Comparing these data (Table 5), it is clear that Lewis acidity of the solvate ILs is higher than the benchmark ionic liquid. However, comparison with the literature data is more difficult, as the authors used an alternative methodology: AN values were recorded not for neat ILs, but for solutions in *d*_6_-benzene, and a large molar ratio of TEPO to IL (1:3) was employed. For comparison, Schmeisser et al. reported AN values for several [C_*n*_mim][NTf_2_] ionic liquids, tested neat, and they were within the range of AN = 25.0–27.4 [24]. AN values measured for chlorometallate ILs are also higher, reaching up to AN = 105 for strongly Lewis acidic compositions (Fig. 3). Possibly, studied neat, AN values for solvate ILs would have been higher.

#### Applications

**Fig. 16:**
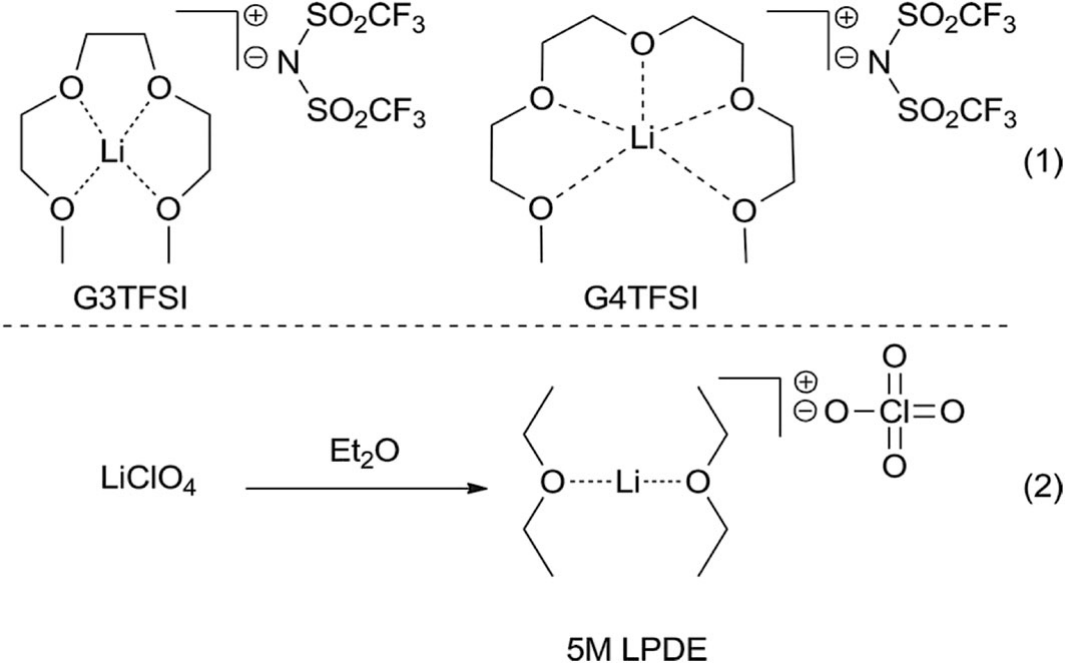
Comparison of the structure of solvate ILs, [Li(G3)][NTf_2_] and [Li(G4)][NTf_2_], and a simplified structure of 5 M LPDE [114]

To date, a single application as a Lewis acidic catalyst was reported by Henderson and co-workers, with [Li(G3)][NTf_2_] and [Li(G4)][NTf_2_] used as solvents and catalysts for Diels–Alder [4 + 2] and for [2 + 2] cycloadditions. They performed better, and were safer and easier to use, than the benchmark 5 M ether solution of lithium perchlorate (Fig. 16) [114]. This result is in agreement with the studied on Lewis acidity of solvate ILs: lower hapticity ligands, such as diethyl ether, make ‘poor ILs’ [176], therefore have lower *α* values, which corresponds to a lower Lewis acidity [187].

### Borenium ILs (Group 13 Lewis Acids)

#### Historical Context

Tricoordinate, molecular boron compounds (boranes, boron halides) are commonly used Lewis acids, their acidity derived from the empty *p*-orbital situated on boron. Lewis acidity depends on substituents; for example, in boron halides it increases in the order BF_3_ < BCl_3_ < BBr_3_ < BI_3_, which is related to decreasing π-back donation from halide to *p*-orbital on the boron, as atomic size of the halide increases [3]. Another strategy to increase electrophilicity relies on introducing a positive charge on the boron center. Tricoordinate borenium cations are stronger Lewis acids compared to structurally similar boranes, having both a net positive charge and a formally vacant *p*-orbital [28].

**Scheme 3:**
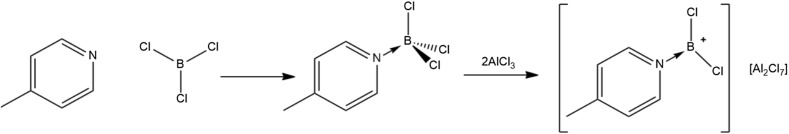
Synthesis of a borenium ionic liquid via chloride abstraction [191]

**Scheme 4:**
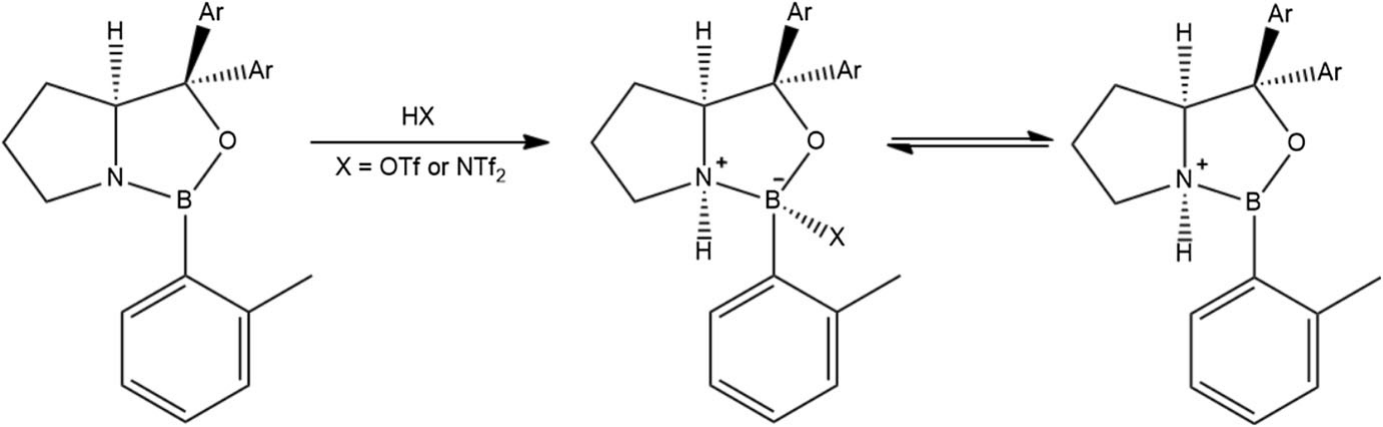
Synthesis of a borenium salt via protonation with a strong Brønsted acid [192]

Synthetic strategies leading to borocations include using aromatic donors to disperse and stabilize charge, and introducing weakly coordination anions, such as [Al_2_Cl_7_]^−^ or [NTf_2_]^−^ (viz. examples in Schemes 3 and 4)—which lends itself to be translated to IL realm. However, despite increasing interest in these strong Lewis acids in main-group and organic community [28, 188, 189], their uses in ILs are both very recent and very rare [115, 190].

#### Speciation

In 2015, Swadźba-Kwaśny and co-workers reported on a range of Lewis acidic ILs synthesized from tetracoordinate boron adducts, [BCl_3_L], and one or two moles of MCl_3_ (M = Al or Ga) [190]. Synthetic procedure was analogous to that of Ryschkevitsch and Wiggins (Scheme 3) [191], albeit the second step (metal chloride addition) was carried out solventless. Ligands (L) included pyridine (py), 3-picoline (3pic), 4-picoline (4pic), 1-methylimidazol (mim), trioctylphosphine (P_888_) and trioctylphosphine oxide (P_888_O).

Upon the addition of MCl_3_ (M = Al, Ga), the ^11^B NMR signal shifted from 5 to 10 ppm (characteristic of four-coordinate boron) to 30–50 ppm, which suggested the formation of a tricoordinate species. In addition to the main broad signal, a sharp peak/shoulder, appearing consistently at 45 ± 1 ppm suggested the presence of molecular BCl_3_, due to ligand transfer to the metal center (Eq. 20). The percentage of ligand transfer was relatively small, with the exception of P_888_O systems, especially combined with AlCl_3_. In this case, where virtually all ligand was transferred to the metal center—thus making an LCC, P_888_O–MCl_3_, with dissolved BCl_3_, rather than a borenium IL.

20 $$\left[ {{\text{BCl}}_{2} {\text{L}}} \right]\left[ {{\text{MCl}}_{4} } \right] \rightleftarrows {\text{BCl}}_{3} + \left[ {{\text{MCl}}_{3} {\text{L}}} \right] \rightleftarrows {\text{BCl}}_{3} + 0.5\left[ {{\text{MCl}}_{2} {\text{L}}_{2} } \right]\left[ {{\text{MCl}}_{4} } \right]$$

In summary, ILs of nominal general formulas [BCl_2_L][MCl_4_] and [BCl_2_L][M_2_Cl_7_] were formed for L = phosphine or aromatic amine, and M = Al or Ga. All these contained some equilibrated BCl_3_ (Eq. 20), therefore notation l-BCl_3_-*n*MCl_3_ was adopted, to avoid oversimplification.

#### Acidity Measurements

**Fig. 17:**
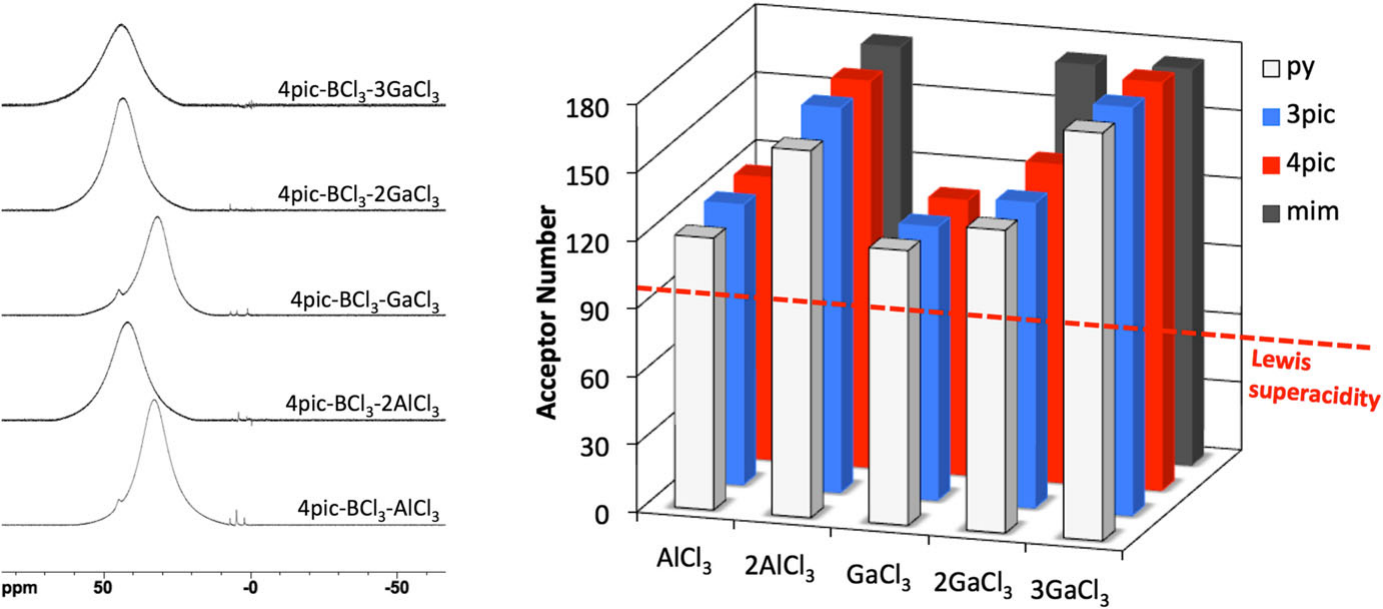
*Left* ^11^B NMR spectra (128.37 MHz, 27 °C, neat liquid with d_6_-DMSO lock) of borenium ILs (composition given on each spectrum); *right* AN values measured for borenium ILs of a general formula l-BCl_3_-*n*MCl_3_, for four different ligands (L), two metals (M) and *n* = 1–3. Adapted from [190]

In ^11^B NMR spectra of borenium ILs, signals from cations with [MCl_4_]^−^ counterions were more shielded than those from cations with [M_2_Cl_7_]^−^ counterions (Fig. 17, left) [190]. This indicates stronger cation–anion interaction for mononuclear [MCl_4_]^−^, compared to dinuclear [M_2_Cl_7_]^−^, and is in agreement with observations for halometallate ILs [14, 58], and with studies on borenium cations in solutions [28].

AN values were measured for all ILs of a general formula l-BCl_3_-*n*MCl_3_, where L = *N*-donor. All ILs were found to be Lewis superacids (AN >100), with some systems reaching AN >180—the highest values reported in the literature [190]. ILs with [MCl_4_]^−^ anions had lower acidities than these with oligomeric anions, in particular [Al_2_Cl_7_]- and [Ga_3_Cl_10_]^−^ (Fig. 17, right), indicating that non-coordinating oligonuclear anions result in the existence of ‘naked’, very acidic borenium cations. Finally, it was shown that AN values recorded for neat IL were higher than those recorded for a solution of this liquid in a molecular solvent.

#### Applications

**Fig. 18:**
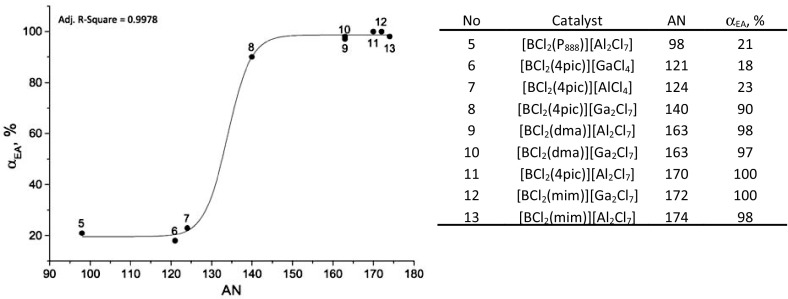
*Left* Conversions in Diels–Alder cycloaddition of cyclopentadiene to ethyl acrylate, catalyzed by 0.10 mol% of borenium ILs *vs*. AN values of the catalysts; *right* tabulated data listing the formula of each IL catalyst, their corresponding ANs and conversions in Diels–Alder reaction. Adapted from [115]

A single application of borenium ILs has been reported to date: as Lewis acidic catalysts in Diels–Alder cycloaddition [115]. Their catalytic performance was correlated with AN values through a sigmoidal curve (Fig. 18), i.e., there was a cut-off point for which a sudden increase in conversion was noted, which is characteristic of Lewis acid-catalyzed reactions [85]. Using ILs with the higher ANs, full conversions were achieved within 5 min at 0.1 mol% of catalyst loading, which surpassed all Lewis acidic ILs previously studied in this reaction (viz. Sect. 2.3.3) [75, 114–117].

Considering the immense catalytic potential of such strong acids, new developments in this area are to be expected, particularly in generating ILs with non-coordinating, non-halometallate anions, thereby eliminating the equilibrium presented in Eq. 20.

## Conclusions

All Lewis acidic ILs discussed in this chapter are characterized by complex speciation, which relates directly to their Lewis acidity and catalytic potential. Therefore, speciation studies—preferably using several in situ techniques—are indispensable to understanding and optimizing the catalytic system. Furthermore, quantifying the Lewis acidity of ILs is a very challenging task. Like in all Lewis acids, the measurement is probe-dependent, therefore order of strength on Lewis acidity scale may alter, depending on the probe used and the methodology adopted.

After being overshadowed by ‘air- and water-stable ILs’, we are witnessing the renaissance of interest in Lewis acidic halometallate ILs, from industrial processes to exciting inorganic synthesis. Liquid coordination complexes (LCCs), which to an extent evolved from halometallate ILs, are of increasing interest. They are expected to gain significantly more attention in the future, due to their higher tunability and lower price.

Finally, the first examples of ILs based on cationic main-group Lewis acids were developed in recent years. This is an entirely new direction of research into Lewis acidic ILs, and the one that potentially can bring the most innovation in the upcoming decade—but also the most synthetic challenges.
